# Effects of Agomelatine on Sleep Across Populations: A Systematic Review and Meta‐Analysis

**DOI:** 10.1111/jsr.70231

**Published:** 2025-11-02

**Authors:** Anastasios Stefanou, Ioannis Anastasiou, Panagiota Fallon, Eleni Glarou, Nikolaos Christodoulou, Andreas S. Lappas, Vasilios‐Panteleimon Bozikas, Myrto T. Samara

**Affiliations:** ^1^ Second Department of Psychiatry, Department of Medicine, School of Health Sciences Aristotle University of Thessaloniki Thessaloniki Greece; ^2^ Medical School, University of Thessaly Larisa Greece; ^3^ Centre for Trials Research Cardiff University Cardiff UK; ^4^ Division of Population Medicine School of Medicine, Cardiff University Cardiff UK; ^5^ Department of Psychiatry School of Medicine, University of Thessaly Larissa Greece; ^6^ Department of Geriatric Psychiatry Aneurin Bevan University Health Board Newport UK

**Keywords:** agomelatine, meta‐analysis, placebo, sleep, sleep quality, systematic review

## Abstract

Agomelatine, a melatoninergic antidepressant, is often prescribed to improve sleep disturbance, though meta‐analytic evidence is currently lacking. This systematic review and meta‐analysis assessed its efficacy and tolerability in sleep outcomes compared to placebo. We systematically searched clinical trial registries (Cochrane Central, WHO ICTRP, ClinicalTrials.gov) and databases (MEDLINE, Embase, APA PsycINFO) up to February 16, 2025, for Randomised Controlled Trials (RCTs) comparing agomelatine with placebo that reported sleep‐related outcomes. Analyses were conducted using a random‐effects model on an intention‐to‐treat basis. Risk ratios (RR) were used for dichotomous outcomes, weighted mean differences (WMD) for continuous outcomes, and Hedge's adjusted g (SMD) when different scales were used. Primary outcomes included subjective and objective total sleep time, subjective sleep quality, and treatment‐emergent somnolence and insomnia. Subgroup and sensitivity analyses explored heterogeneity and assessed robustness. Twenty‐five RCTs with 6812 participants were included. No significant effect was found for objective total sleep time (MD = −15.73 min, 95% CI: −49.68; 18.22), while subjective sleep quality improved more with agomelatine than placebo (SMD = 0.31, 95% CI: 0.21; 0.40). Agomelatine was associated with fewer incidents of insomnia (RR = 0.59, 95% CI: 0.39; 0.90) but more incidents of somnolence (RR = 1.34, 95% CI: 1.02; 1.75). Agomelatine was found to cause marginally more adverse effects than placebo (RR = 1.05, 95% CI: 1.00; 1.11). Overall, agomelatine appears to slightly improve sleep quality and is well‐tolerated and safe, although the limited data for many outcomes warrant cautious interpretation.

## 1 Introduction

Sleep is considered a vital aspect for an individual's functionality, thus not only sleep quantity but also the quality is of high significance (Zielinski et al. 2016). Sleep problems are highly prevalent in the general population and are associated with various medical conditions (Medic et al. 2017), such as cardiovascular (Meier‐Ewert et al. 2004) and metabolic comorbidities (Gottlieb et al. 2005). Additionally, several psychiatric disorders negatively impact sleep architecture, further disrupting mental and cognitive functions (e.g., mood, concentration) (Chattu et al. 2019; Sejbuk et al. 2022). On that note, there is growing evidence supporting a bidirectional causal link between sleep problems and psychiatric (e.g., Major Depressive Disorder) (Fang et al. 2019) and neurodegenerative disorders (e.g., Alzheimer's Disease) (Astara et al. 2024; Krystal 2012; Wang and Holtzman 2020). Sleep problems can impair individuals' daily and socio‐professional functioning, along with their overall quality of life (Katz and McHorney 2002; Léger et al. 2002), resulting in high service utilisation and socioeconomic costs (Streatfeild et al. 2021).

Sleep problems and insomnia are increasingly recognised as symptoms transcending specific diagnoses. Major diagnostic systems—the International Classification of Sleep Disorders‐3rd edition (ICSD‐3) (Sateia 2014), Diagnostic and Statistical Manual of Mental Disorders—5th edition (DSM‐V) (American Psychiatric Association 2013), and International Statistical Classification of Diseases and Related Health Problems—11th revision (ICD‐11)(World Health Organization 2019) – have eliminated the distinction between primary and secondary insomnia, reflecting a consensus that chronic insomnia constitutes a distinct disorder warranting direct treatment regardless of aetiology or comorbidity. Consequently, clinical guidelines emphasise addressing insomnia as an independent therapeutic target, irrespective of its co‐occurrence with other disorders (Riemann et al. 2017; Thorpy 2017), with first‐line treatments for insomnia (i.e., CBT‐I) showing effectiveness (Geiger‐Brown et al. 2015; Zhou et al. 2020), regardless of aetiology or comorbidities.

Despite this unified approach, treating insomnia clinically remains challenging (Samara 2022) and current treatment guidelines only refer to specific diagnoses such as insomnia disorder (Sateia et al. 2017), creating a significant implementation gap. While aetiology does not change treatment principles, comorbidities complicate intervention by reducing patient capacity for standard therapies (Agnew et al. 2021; Lawson et al. 2023; Nijhof et al. 2024) and increasing medication interaction risks (Marović et al. 2024). This often delays or undermines insomnia‐specific treatment even when indicated.

Effective antidepressants are known to improve disturbed sleep, as well as the circadian cycle, such as the sleep/wake cycle's rhythms of depressive disorders (Pandi‐Perumal et al. 2009; Tsuno et al. 2005). Additionally, they may reduce the nocturnal awakening frequency as well as latency to sleep onset, while also boosting alertness in daytime (Tchekalarova et al. 2020). However, the majority of antidepressants do not restore sleep architecture, and patients receiving selective serotonin reuptake inhibitors (SSRIs) are also treated with benzodiazepines and/or hypnotics (e.g., zolpidem) (Rascati 1995; Thase 2006) to mitigate various sleep disorders, such as insomnia (Bushnell et al. 2022; Scharner et al. 2022). Nevertheless, these medications often come with several adverse effects of various severity, including nausea, headache, dyspepsia, even leading to addiction, cognitive disruption and driving underperformance (Gunja 2013; Jung et al. 2020; Lucchetta et al. 2018).

Agomelatine, approved in Europe since 2009 (Servier Laboratories 2009b), is administered to treat mood and anxiety disorders (e.g., Major Depressive Disorder [MDD] and/or Generalised Anxiety Disorder [GAD]) as per its main indications (Guaiana et al. 2013). It constitutes a novel antidepressant acting as an agonist on the melatonin receptors (MT1 and MT2) and an antagonist on the serotonin receptors (5‐HT2C and 5‐HT2B) (Zupancic and Guilleminault 2006).

Although sleep disturbances are among the first symptoms to show improvement with agomelatine in patients with MDD (Stahl 2021b), most systematic reviews and meta‐analyses on agomelatine have focused on its efficacy, safety, and tolerability in treating symptoms of depression, without further elaborating on sleep behaviour (Guaiana et al. 2013; Guo et al. 2023; Koesters et al. 2013; Taylor et al. 2014). While some evidence suggests that off‐label use of agomelatine may improve sleep in conditions beyond depression (De Berardis et al. 2015), recent large‐scale network meta‐analyses comparing various treatments for insomnia or sleep disturbances in a range of psychiatric disorders have not included agomelatine (Crescenzo et al. 2022; Lappas, Glarou, et al. 2024; Lappas, Polyzopoulou, et al. 2024; Samara et al. 2020).

Despite advancements in insomnia diagnosis and classification, significant gaps persist in treatment evidence. This systematic review and meta‐analysis addresses these by synthesising and appraising all available RCT evidence on the effects of agomelatine versus placebo on sleep parameters irrespective of primary or comorbid medical conditions.

## 2 Methods

This review was conducted according to the Preferred Reporting Items for Systematic Reviews and Meta‐analyses (PRISMA) statement (Table S1) (Moher et al. 2009).

### 2.1 Protocol

An a priori written study protocol (CRD42022385063) was published in PROSPERO in December 2022 and is provided in detail in the Supporting Information Section 2.

### 2.2 Population, Intervention, and Types of Included Studies

Patients with any type of health problems were included, irrespective of any psychiatric or medical diagnosis, not excluding healthy individuals. No restrictions in terms of age, sex, ethnicity, comorbidities, chronicity of illness, dose range, or system of diagnostic classification were applied. The intervention of interest was agomelatine, administered in any dose, form or preparation (e.g., oral tablets, sublingual administration), either as monotherapy or as augmentation to any other treatment, compared to placebo. The studies' eligibility criteria included: (a) inclusion of only RCTs (excluding cluster RCTs); (b) no restrictions on blinding methods, accepting open‐label, single‐blind, or double‐blind designs; (c) a minimum pharmacotherapy duration of at least 5 days, based on previous meta‐analytic research on sleep (Samara et al. 2020); and (d) reporting of any sleep‐related efficacy, safety, or tolerability outcomes.

### 2.3 Outcome Measures

#### 2.3.1 Primary Outcomes

The primary outcomes of our study were (i) subjective total sleep time (continuous) measured in minutes; if no studies included this measurement, we would report objective total sleep time, if available (ii) sleep quality (continuous) as measured by any validated sleep quality measure/questionnaire, such as Pittsburgh sleep quality index (PSQI) or Leeds sleep evaluation questionnaire (LSEQ); (iii) number of participants with somnolence (dichotomous) as a treatment emergent side effect and (iv) number of participants with insomnia (dichotomous) as a treatment emergent side effect.

#### 2.3.2 Secondary Outcomes

Our review also included the following secondary outcomes: (i) subjective sleep onset latency (SOL) (continuous), that is, the time needed to fall asleep, which serves as an indicator of sleep onset insomnia; (ii) objective SOL, measured through polysomnography or actigraphy; (iii) subjective number of nocturnal awakenings (NAw) (continuous), representing disturbances in sleep continuity; (iv) objective NAw measured through polysomnography or actigraphy; (v) subjective nocturnal time spent awake after sleep onset (WASO) (continuous), a quantitative measure of sleep maintenance; (vi) objective WASO, measured through polysomnography or actigraphy; (vii) daytime impairment (DI) (continuous), assessed through performance tasks and self‐reported scales like the Epworth Sleepiness Scale or the Stanford Sleepiness Scale; (viii) patients' subjective well‐being/quality of life (e.g., SF‐36, EURO‐Qol) (continuous), an outcome that integrates aspects of both efficacy and tolerability; (ix) polysomnographic or actigraphic recordings of the primary outcome, ‘total nocturnal sleep time’ (TST‐PSG) (continuous), enabling the exploration of potential differences between patient‐rated subjective and clinician‐rated objective evaluations of insomnia; (x) number of participants reporting parasomnias (dichotomous), specifically nightmares, vivid dreams and parasomnia behaviours; (xi) number of dropouts due to adverse effects (dichotomous); (xii) number of dropouts due to sleep‐related adverse effects (dichotomous); (xiii) number of participants with adverse effects as a global measure of tolerability (dichotomous); (xiv) number of participants with sleep‐related adverse effects (dichotomous); and (xv) the number of participants who required hypnotic rescue treatment for insomnia using a hypnotic drug other than agomelatine, as required during the trial (dichotomous); and (xvi) any other relevant outcomes, such as behaviour integrity, as a perceived impact of sleep on cognitive and psychomotor functioning upon waking.

### 2.4 Search Strategies, Selection Criteria and Data Extraction

A systematic literature search was undertaken using Medline (via Ovid – see Table S2 for search string), EMBASE, APA (American Psychological Association, via PsycINFO), Cochrane Central Register of Controlled Trials (CENTRAL), http://clinicaltrials.gov, the WHO International Clinical Trials Registry Platform (ICTRP) up to February 16, 2025. No limitations were applied in terms of language, year, and status of publication. We also searched and screened the references of previously published relevant reviews and all included studies if applicable.

At least two reviewers (AS, IA, PF, and EG) independently screened all abstracts and subsequently the relevant full texts from the searches performed, as well as additional records identified through other sources. This process was conducted using Rayyan, a web‐based tool designed to assist researchers in systematic reviews (Ouzzani et al. 2016). Any conflicts that arose during the selection process were resolved through extensive discussions among the reviewers and, when necessary, with the senior authors (MS and AL).

Data extraction was performed by two reviewers (IA and PF) independently using the same a priori standardised data extraction spreadsheets. The first and/or corresponding authors from all included studies were contacted for missing information and possible corrections. In case of missing data concerning standard deviation (SD), respective values were calculated through standard errors, confidence intervals (CIs) and *p*‐values based on the formulas provided by Cochrane (Higgins et al. 2023) or, in some cases, were imputed by the mean SD of other studies (Furukawa et al. 2006). Finally, any conflict between the reviewers was resolved through discussion with the senior authors (AL and MS).

### 2.5 Statistical Analysis

This meta‐analysis was conducted with the use of R Studio version 4.4.1 (R Core Team 2024). Endpoint values were considered preferable over change values to abstain from missing information, given the limited availability and/or quality of the change data, often due to missing SDs; however, post‐intervention values do not account for baseline imbalances and may lack statistical power (Deeks et al. 2024). We employed the random‐effects model of meta‐analysis. The model accounts for between‐study variability and yields wider CIs; thus, it is typically more conservative in assessing statistical significance (Borestein et al. 2009). However, a potential drawback is that it assigns more weight to smaller studies, which can either inflate or deflate the effect size (Dettori et al. 2022). To test the robustness of our findings, we performed a sensitivity analysis for the primary outcomes, examining the effect of using a fixed‐effects model.

For dichotomous outcomes risk ratio (RR) was calculated, while weighted mean difference (WMD) was used for continuous variables. When an outcome had different units of measurement, the effect size was calculated as Hedge's adjusted g (standardised mean difference, SMD). Effect sizes are presented along with their 95% CIs, calculated based on the standard error of the mean. We also present respective prediction intervals (PIs), which incorporate between‐study heterogeneity and reflect the range of effects in future similar studies (Borestein et al. 2009).

#### 2.5.1 Heterogeneity, Subgroup and Sensitivity Analyses

Heterogeneity was assessed with the *I* ^*2*^‐value and its *p*‐value.

Subgroup analyses were performed for all primary outcomes. The following subgroups were considered a priori, (depending on data availability): (a) per primary diagnosis, (b) participants with sleep disturbance symptoms versus not, (c) monotherapy versus add‐on agomelatine treatment, (d) participants older than 65 versus not., (e) comorbid substance misuse versus not., (f) presence of an organic mental disorder versus not., and (g) presence of a primary medical disorder versus not.

Sensitivity analyses for primary outcomes were also planned a priori: (a) exclusion of non‐double–blind studies (open and single‐blind studies), (b) exclusion of studies that presented only completer analyses, (c) exclusion of studies with high risk of bias, (d) fixed effect instead of random effects model, (e) exclusion of studies with imputed data, (f) exclusion of studies sponsored by industry, and (g)exclusion of studies that allowed the use of hypnotics other than agomelatine which were prescribed as required during the study.

### 2.6 Risk of Bias

At least two independent reviewers (EG, IA, and PF) assessed the risk of bias using the Cochrane risk of bias tool (study based) for randomised trials (RoB) (Higgins et al. 2011). The overall risk of bias for each study was classified as ‘high,’ ‘moderate,’ or ‘low’ based on the assessment of individual risk of bias components according to Furukawa et al. (Furukawa et al. 2016) (Table S4).

### 2.7 Publication Bias

To address potential publication bias, our search strategy included grey literature databases, such as clinical trial registries and major conferences' abstract lists (see paragraph 2.3). For the primary outcomes, funnel plots with a minimum of 10 studies (Higgins and Green 2011) were generated and evaluated for symmetry, using the ‘trim and fill’ method (Duval and Tweedie 2000) and the Egger's *g* test (Egger et al. 1997).

## 3 Results

### 3.1 Search Results and Characteristics of Included Studies

We identified 25 relevant RCTs with a total of 6812 randomised participants. The studies were published between 2002 and 2024. The PRISMA flow diagram (Page et al. 2021) and table of included studies are presented in the Supporting Information (Figure S1 and Table S3, respectively). The mean number of patients per study was 272 and the median was 228. The range of the sample size per study was between 16 and 711 patients. All 25 employed double‐blind design, while two RCTs (Ballester et al. 2019; Leproult et al. 2005) used a crossover‐randomisation method. Many of the RCTs were conducted in Finland (11 studies, 44% of all included studies). Of the 25 studies, 20 RCTs compared agomelatine with placebo as monotherapies, while five studies (Arango et al. 2022; Azadi et al. 2024; Mahdavi et al. 2022; Shokrani et al. 2023; Yatham et al. 2016) examined both agomelatine and placebo as adjuncts to escitalopram, lithium, pregabalin, sertraline, valproate or psychosocial counselling. The majority of the studies included adults; mean age was 42.24 years (range 18–82). One study (Heun et al. 2013) involved elderly patients exclusively, and another study (Arango et al. 2022) examined children and adolescents, aged < 18 years. Females comprised a larger proportion of the population (61.23%) and were consistently predominant in almost all studies, besides six (Ballester et al. 2019; Leproult et al. 2005; Lôo et al. 2002; Mahdavi et al. 2022; Nejati et al. 2024; Zohar and Servier Laboratories 2009). One study (Salin et al. 2019) did not provide any demographics. The majority of the included RCTs were sponsored (72%), with the exception of seven studies (Azadi et al. 2024; Kennedy and Emsley 2006; Lôo et al. 2002; Mahdavi et al. 2022; Nejati et al. 2024; Shokrani et al. 2023; Stein et al. 2008). We imputed SDs in six studies (Ballester et al. 2019; Leproult et al. 2005; Novartis Pharmaceuticals 2020; Salin et al. 2019; Stahl et al. 2010; Zajecka et al. 2010).

The 25 relevant RCTs involved participants with a wide spectrum of diagnoses, including: (a) Major Depressive Disorder (MDD) (12/25) (Arango et al. 2022; Azadi et al. 2024; Heun et al. 2013; Kennedy and Emsley 2006; Kennedy et al. 2014; Lôo et al. 2002; Novartis Pharmaceuticals 2020; Olié and Kasper 2007; Rouillon and Servier Laboratories 2008; Servier Laboratories 2009a; Stahl et al. 2010; Zajecka et al. 2010), (b) Generalised Anxiety Disorder (GAD) (4/25) (Stein et al. 2008, 2012, 2014, 2017), (c) Obsessive Compulsive Disorder (OCD) (3/25) (Nejati et al. 2024; Shokrani et al. 2023; Zohar and Servier Laboratories 2009), (d) Autism Spectrum Disorder (ASD) (1/25) (Ballester et al. 2019), (e) Bipolar Disorder type I (1/25) (Yatham et al. 2016), (f) Systemic Lupus Erythematosus (1/25) (Salin et al. 2019), (g) Chronic Low Back Pain (1/25) (Mahdavi et al. 2022), while (h) two RCTs included healthy participants (2/25) (Leproult et al. 2005; Montejo et al. 2015). For all psychiatric diagnoses, the respective diagnostic criteria were used by the authors: DSM‐IV criteria for MDD, GAD, OCD, and Bipolar Disorder Type I, and DSM‐5 criteria for ASD.

A total of 11 ongoing studies were identified and provided in detail in the Supporting Information (Table S5).

### 3.2 Risk of Bias Assessment

A total of 18 studies (72%) were judged as having an overall low risk of bias, while seven studies (28%) were judged as having an overall moderate risk of bias. The risk of bias summary plot and assessment per individual study are presented in the Supporting Information (Figures S2 and S3, respectively).

### 3.3 Primary Outcomes

#### 3.3.1 Total Sleep Time (TST)

##### 3.3.1.1 Subjective Total Sleep Time

No study reported subjective TST.

##### 3.3.1.2 Objective Total Sleep Time

**FIGURE 1:**
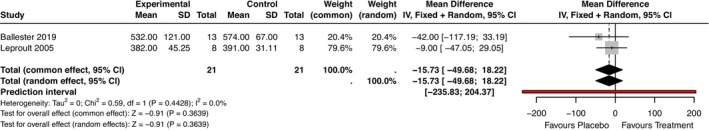
Forest plot—Total sleep time measured in minutes, pooled result. MD = Weighted Mean Difference for TST with 95% CI (Confidence Intervals) and PI (Prediction Intervals).

Only two studies provided objective TST data (Ballester et al. 2019; Leproult et al. 2005). Ballester et al. 2019 measured TST using the Ambulatory Circadian Monitoring, while Leproult et al. (2005) used Polysomnography. The results of both studies were quantified in minutes. No difference between agomelatine and placebo was found (MD = −15.73 min, 95% CI: −49.68; 18.22, *p*‐value = 0.36, PI: −235.83; 204.37, two RCTs, *N* = 42, I^2^ = 0%, Figure 1).

#### 3.3.2 Quality of Sleep

**FIGURE 2:**
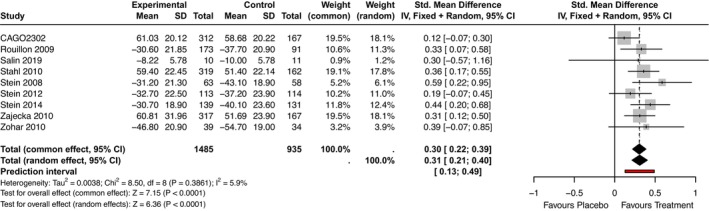
Forest plot—Quality of Sleep measured in LSEQ or PSQI, pooled result. SMD = standardised mean difference for Quality of Sleep (measured in LSEQ or PSQI) with 95% CI (Confidence Intervals) and PI (Prediction Intervals).

Nine studies reported outcomes on the quality of sleep using either the LSEQ or the PSQI (Novartis Pharmaceuticals 2020; Rouillon and Servier Laboratories 2008; Salin et al. 2019; Stahl et al. 2010; Stein et al. 2008, 2012, 2014; Zajecka et al. 2010; Zohar and Servier Laboratories 2009). The meta‐analysis showed that agomelatine improved the overall quality of sleep for participants compared to placebo (SMD = 0.31, 95% CI: 0.21; 0.40, *p*‐value < 0.01, PI: 0.13; 0.49, nine RCTs, *N* = 2420, I^2^ = 5.9%, Figure 2).

#### 3.3.3 Insomnia as Treatment Emergent Side Effect

**FIGURE 3:**
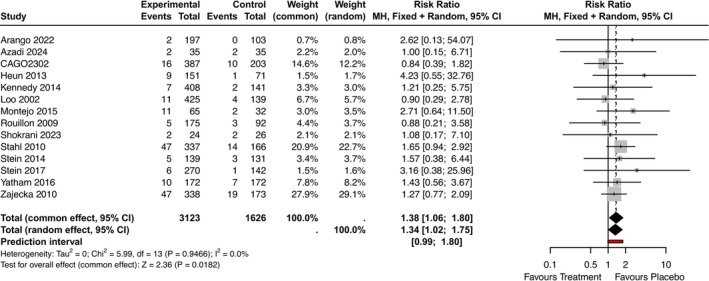
Forest plot—Insomnia as a treatment emergent side effect, pooled result. RR = Risk Ratio for Insomnia as treatment emergent adverse effect with 95% CI (Confidence Intervals) and PI (Prediction Intervals).

Seven studies reported insomnia as a treatment‐emergent side effect (Kennedy et al. 2014; Kennedy and Emsley 2006; Lôo et al. 2002; Novartis Pharmaceuticals 2020; Shokrani et al. 2023; Yatham et al. 2016; Zajecka et al. 2010). The analysis showed that fewer participants on agomelatine experienced insomnia compared to those on placebo (RR = 0.59, 95% CI: 0.39; 0.90, *p*‐value = 0.01, PI: 0.35; 1.00, seven RCTs, *N =* 2835, I^2^ = 0%, Figure 3).

#### 3.3.4 Somnolence as a Treatment Emergent Side Effect

**FIGURE 4:**
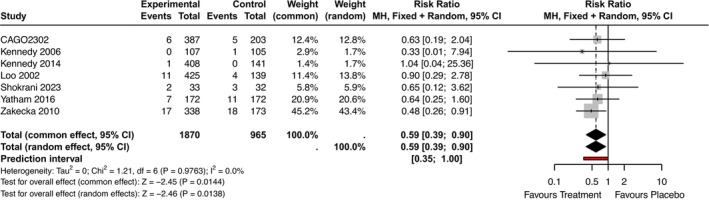
Forest plot—Somnolence as a treatment emergent side effect, pooled result. RR = Risk Ratio of Somnolence as treatment emergent adverse effect with 95% CI (Confidence Intervals) and PI (Prediction Intervals).

A total of 14 RCTs reported somnolence as a treatment‐emergent side effect (Arango et al. 2022; Azadi et al. 2024; Heun et al. 2013; Kennedy et al. 2014; Lôo et al. 2002; Montejo et al. 2015; Novartis Pharmaceuticals 2020; Rouillon and Servier Laboratories 2008; Shokrani et al. 2023; Stahl et al. 2010; Stein et al. 2014, 2017; Yatham et al. 2016; Zajecka et al. 2010). The results showed that more participants on agomelatine experienced somnolence compared to placebo (RR = 1.34, 95% CI: 1.02–1.75, *p*‐value = 0.04, PI: 0.99–1.80, 14 RCTs, *N* = 4749, I^2^ = 0%, Figure 4).

### 3.4 Secondary Outcomes

#### 3.4.1 Sleep Onset Latency

##### 3.4.1.1 Subjective Sleep Onset Latency (Getting to Sleep Score Measured in LSEQ)

Eight RCTs reported sleep onset latency using the ‘getting off to sleep’ LSEQ sub‐score (Novartis Pharmaceuticals 2020; Rouillon and Servier Laboratories 2008; Stahl et al. 2010; Stein et al. 2008, 2012, 2014; Zajecka et al. 2010; Zohar and Servier Laboratories 2009). The meta‐analysis showed that agomelatine decreases sleep onset latency compared to placebo (SMD = −0.28, 95% CI: −0.53; −0.03; *p*‐value = 0.03, PI: −1.12–0.56, eight RCTs, *N* = 2388, I^2^ = 89.3, Figure S8.1).

##### 3.4.1.2 Objective Sleep Onset Latency

Two RCTs reported sleep onset latency measured in minutes (Ballester et al. 2019; Leproult et al. 2005). Ballester et al. (Ballester et al. 2019) measured it using the Ambulatory Circadian Monitoring, while Leproult et al. (Leproult et al. 2005) used Polysomnography. This analysis showed that agomelatine does not significantly decrease sleep onset latency compared to placebo. (MD = 1.48 min, 95% CI: −11.02; 13.97; *p*‐value = 0.82, PI: −79.53; 82.48, two RCTs, *N* = 42, I^2^ = 0.0, Figure S8.2).

#### 3.4.2 Number of Nocturnal Awakenings

##### 3.4.2.1 Subjective Number of Nocturnal Awakenings

No study reported the subjective number of nocturnal awakenings.

##### 3.4.2.2 Objective Number of Nocturnal Awakenings

Two RCTs reported the objective number of nocturnal awakenings (Ballester et al. 2019; Leproult et al. 2005). Ballester et al. (Ballester et al. 2019) measured this outcome using the Ambulatory Circadian Monitoring, while Leproult et al. (Leproult et al. 2005) used Polysomnography. The results showed that there is no difference between agomelatine and placebo (MD = 0.70 times, 95% CI: −0.82; 2.22, *p*‐value = 0.37, PI: −9.16; 10.55, two RCTs, *N* = 42, I^2^ = 0%, Figure S9).

#### 3.4.3 Nocturnal Time Spent Awake After Sleep Onset

##### 3.4.3.1 Subjective Nocrutnal Time Spent Awake After Sleep Onset

No study reported subjective wakefulness after sleep onset.

##### 3.4.3.2 Objective Nocrutnal Time Spent Awake After Sleep Onset

Two RCTs reported objective nocturnal time spent awake after sleep onset (Ballester et al. 2019; Leproult et al. 2005). Ballester et al. (Ballester et al. 2019) measured the nocturnal time spent awake after sleep onset using the Ambulatory Circadian Monitoring, while Leproult et al. (Leproult et al. 2005) used Polysomnography. The results showed that there is no difference between agomelatine and placebo, concerning this outcome (MD = 12.22 min, 95% CI: −13.90; 38.34, *p*‐value = 0.36, PI: −157.12; 181.56, two RCTs, *N* = 42, I^2^ = 0%, Figure S10).

#### 3.4.4 Daytime Impairment (Sleep Awakening Score)

Six RCTs reported daytime impairment, using the ‘Sleep awakening’ LSEQ sub‐score to report on daytime impairment of participants (Novartis Pharmaceuticals 2020; Rouillon and Servier Laboratories 2008; Stein et al. 2008, 2012, 2014; Zohar and Servier Laboratories 2009). The meta‐analysis showed no statistically significant improvement in daytime impairment by agomelatine compared to placebo (SMD = 0.19, 95% CI: −0.01; 0.38; *p* = 0.06, PI: −0.38; 0.75, six RCTs, *N* = 1435, I^2^ = 62.2%, Figure S11).

#### 3.4.5 Number of Dropouts Due to Adverse Effects

A total of 22 RCTs reported the number of dropouts due to any adverse effect (Arango et al. 2022; Ballester et al. 2019; Heun et al. 2013; Kennedy et al. 2014; Kennedy and Emsley 2006; Lôo et al. 2002; Mahdavi et al. 2022; Montejo et al. 2015; Novartis Pharmaceuticals 2020; Olié and Kasper 2007; Rouillon and Servier Laboratories 2008; Salin et al. 2019; Servier Laboratories 2009a; Shokrani et al. 2023; Stahl et al. 2010; Stein et al. 2008, 2012, 2014, 2017; Yatham et al. 2016; Zajecka et al. 2010; Zohar and Servier Laboratories 2009). There were no significant differences between agomelatine and placebo groups (RR = 0.89, 95% CI: 0.78–1.02; *p*‐value = 0.09, 22 RCTs, *N* = 6216, I^2^ = 31.6%, Figure S12).

#### 3.4.6 Number of Dropouts Due to Sleep‐Related Adverse Effects

Only one RCT reported the number of dropouts due to sleep‐related adverse effects (Arango et al. 2022) and, therefore, no meta‐analysis was conducted (Figure S13).

#### 3.4.7 Number of Participants With Adverse Effects

A total of 22 RCTs reported the number of participants with adverse effects as a global measure of tolerability (Arango et al. 2022; Azadi et al. 2024; Ballester et al. 2019; Heun et al. 2013; Kennedy et al. 2014; Kennedy and Emsley 2006; Lôo et al. 2002; Montejo et al. 2015; Nejati et al. 2024; Novartis Pharmaceuticals 2020; Olié and Kasper 2007; Rouillon and Servier Laboratories 2008; Servier Laboratories 2009a; Shokrani et al. 2023; Stahl et al. 2010; Stein et al. 2008, 2012, 2014, 2017; Yatham et al. 2016; Zajecka et al. 2010; Zohar and Servier Laboratories 2009). The risk of adverse effects was higher in patients receiving agomelatine compared to placebo (RR = 1.05, 95% CI: 1.00; 1.11, *p*‐value = 0.04, 22 RCTs, *N* = 6253, I^2^ = 16.6%, Figure S14).

#### 3.4.8 Number of Participants With Sleep—Related Adverse Effects

A total of 17 RCTs reported the number of participants with sleep‐related adverse effects (Arango et al. 2022; Heun et al. 2013; Kennedy et al. 2014; Kennedy and Emsley 2006; Lôo et al. 2002; Mahdavi et al. 2022; Montejo et al. 2015; Novartis Pharmaceuticals 2020; Olié and Kasper 2007; Rouillon and Servier Laboratories 2008; Shokrani et al. 2023; Stahl et al. 2010; Stein et al. 2014, 2017; Yatham et al. 2016; Zajecka et al. 2010; Zohar and Servier Laboratories 2009). Risk of sleep‐related adverse effects did not differ significantly between agomelatine and placebo groups (RR = 1.07, 95% CI: 0.88; 1.31, *p*‐value = 0.49, 17 RCTs, *N* = 5264, I^2^ = 0%, Figure S15).

#### 3.4.9 Other Sleep Related Outcomes (Behaviour Integrity)

Four RCTs reported behaviour integrity as a subscale of the LSEQ (Arango et al. 2022; Heun et al. 2013; Kennedy et al. 2014; Kennedy and Emsley 2006; Lôo et al. 2002; Mahdavi et al. 2022; Montejo et al. 2015; Novartis Pharmaceuticals 2020; Olié and Kasper 2007; Rouillon and Servier Laboratories 2008; Shokrani et al. 2023; Stahl et al. 2010; Stein et al. 2014, 2017; Yatham et al. 2016; Zajecka et al. 2010; Zohar and Servier Laboratories 2009). The meta‐analysis showed no improvement by agomelatine compared to placebo, concerning this outcome (SMD = 0.13, 95% CI: −0.00; 0.27, *p*‐value = 0.58, four RCTs, *N* = 1314, I^2^ = 15.5%, Figure S16).

None of the identified RCTs reported the following outcomes: (i) patients' subjective well‐being/quality of life; and (ii) the number of participants reporting parasomnias.

### 3.5 Subgroup and Sensitivity Analyses

Subgroups had, at most, insufficient data and no difference from the pooled results was found. Additionally, the conclusions for the primary outcomes remained consistent and did not change substantially in a series of preplanned sensitivity analyses (Supp. Material, Section 9.1. Primary Outcomes).

### 3.6 Publication Bias

Funnel plots were generated only for one primary outcome, ‘Somnolence as treatment emergent side effect’ (Figures S17 and S18). Egger's test showed no funnel plot asymmetry, but the trim‐and‐fill method showed two missing studies with large standard errors, possibly implying small studies effect (Supp. Material Section 10. ‘Assessment of Publication bias’).

## 4 Discussion

The aim of this systematic review and meta‐analysis was to synthesise all available RCTs that compared agomelatine with placebo, concerning sleep‐related outcomes, regardless of age, sex and/or primary diagnosis. To our knowledge, this was the first meta‐analysis to prioritise sleep parameters as outcomes of interest; it included 25 RCTs and a total of 6812 participants.

Based on our findings, agomelatine was not shown to improve any objective sleep quantity parameters, such as TST, SOL, number of nocturnal awakenings or wakefulness after sleep onset. Subjective SOL and sleep quality were improved by agomelatine, though by a small effect size. Concerning safety, agomelatine was found to cause marginally more treatment‐emergent adverse effects, especially somnolence (RR = 1.34, ΝΝΗ = 67). However, it was generally tolerable, as there was no difference in dropouts due to adverse effects between agomelatine and placebo.

Despite clinical heterogeneity in terms of diagnostic inclusion criteria, statistical heterogeneity was generally low (I^2^‐value < 30%) in the synthesis of all primary outcomes and most secondary outcomes.

Concerning the cumulative sample size, key efficacy outcomes, such as objective TST, sleep onset latency (measured in minutes), number of nocturnal awakenings and WASO, were only reported in two RCTs (*N* = 42) (Ballester et al. 2019; Leproult et al. 2005). Thus, our findings of non‐significance warrant cautious interpretation due to limited statistical power. However, outcomes with larger trial numbers (e.g., sleep quality, LSEQ sleep onset latency and daytime impairment) still suggest only minimal improvement. Crucially, safety outcomes—including treatment‐emergent somnolence, adverse event‐related dropouts, and overall/sleep‐related adverse events—incorporated more RCTs, with sample sizes exceeding the widely accepted threshold of 1000 participants (Trikalinos et al. 2004). This enhances confidence in the results, indicating a favourable safety and tolerability profile for agomelatine.

Agomelatine, as a melatoninergic and serotoninergic antidepressant, may resynchronize altered circadian rhythm, restore the circadian sleep–wake cycle, and thereby improve sleep structure (Bourin and Prica 2009; Liu et al. 2016; Millan 2022; Stahl 2021a; Su et al. 2023). However, preclinical research findings do not translate to clinical (especially those of RCTs, as per the present meta‐analysis).

Furthermore, a disparity between objective and self‐reported outcomes is implied by our results. Even though some studies indicate that self‐reported measures may be in accordance with objective actigraphic findings (Lemola et al. 2013), subjective judgements (e.g., sleep quality) may also be influenced by post‐awakening daily experience, as highlighted in our recent meta‐analysis on trazodone and sleep (Kokkali et al. 2024). In particular, the subjective nature of sleep quality questionnaires and their reference to a long recall period may compromise their reliability (Fabbri et al. 2021).

Concerning objective efficacy outcomes, observational and open‐label studies using polysomnographic and actigraphic records have yielded findings similar to ours on the effects of agomelatine on sleep‐related parameters. In patients with major depressive disorder (MDD), agomelatine has been found to have no effect on TST, though findings on SOL, NAW and WASO were inconclusive (Porteous et al. 2021; Quera Salva et al. 2007). However, real‐world data from observational studies suggest that agomelatine may increase polysomnographic TST and reduce the number of awakenings (Avila et al. 2015; Poluéktov and Levin 2013). Additionally, a single‐blinded RCT in patients with obstructive sleep apnea reported improvements in TST, sleep efficiency, and nocturnal awakenings with agomelatine, though the lack of a placebo control may have introduced performance or attrition bias (Dastan et al. 2023). According to our results, these findings remain largely unexplored and have not yet been confirmed by randomised placebo‐controlled trial evidence.

Further discussion arises from head‐to‐head comparisons with other antidepressants, which have not been included in the present meta‐analysis (Table S6). In general agomelatine has been found comparable to escitalopram, sertraline, fluoxetine, mirtazapine, venlafaxine and duloxetine, both in clinician‐rated and subjective sleep outcomes in patients with MDD and GAD. (Corruble et al. 2013; Kasper et al. 2010; Lemoine et al. 2007; Marey and Servier Laboratories 2020; Mi et al. 2020; Quera‐Salva et al. 2011; Shu et al. 2014; Stein et al. 2018). Therefore, head‐to‐head trials need to be considered and further evaluated in meta‐analytic research.

### 4.1 Limitations

Our study has several limitations. First, although I^2^ values were generally low for most outcomes (< 30%), our broad inclusion criteria (e.g., no restrictions on diagnosis or age) introduced clinical heterogeneity. Importantly, populations like older adults and patients with OCD were underrepresented (*n* = 1 study each), limiting the generalisability of our findings in such populations. Additionally, most sleep‐related efficacy measures were reported in only two RCTs (*N* = 42), resulting in underpowered analyses that may have led to type II errors, meaning that true differences could not be detected due to the small sample size. Most of the studies included were industry‐sponsored and were judged to have a moderate risk of bias, further affecting the reliability of our conclusions. Moreover, the duration of included RCTs, the exclusive focus on placebo‐controlled designs and the inclusion of two crossover trials may have influenced treatment effects and reduced inter‐trial comparability. Also, many included studies did not align with evidence on agomelatine's dose‐dependent efficacy (Kennedy et al. 2014). Specifically, 10 of 25 RCTs used the lowest approved dose of 25 mg/day, but the absence of efficacy data in these trials mitigates concerns about their impact on our analysis. Finally, the reliance on self‐reported sleep measures in several studies introduced the potential for additional bias, emphasising the need for further research incorporating objective sleep assessments.

## 5 Conclusion

In summary, this meta‐analysis suggests that agomelatine may improve self‐reported sleep quality and sleep onset latency and is associated with fewer incidents of insomnia and increased somnolence compared to placebo. However, it showed no effect on other sleep‐related outcomes. These findings should be interpreted with caution, as many outcomes were based on limited data. Further research and comparisons with active controls are urgently needed to determine whether agomelatine is a valuable treatment for insomnia.

## Author Contributions

**Anastasios Stefanou:** writing – original draft, visualization, writing – review and editing, software, formal analysis, investigation. **Ioannis Anastasiou:** investigation, software, formal analysis, writing – original draft, visualization. **Panagiota Fallon:** investigation, writing – original draft, visualization, software, formal analysis. **Eleni Glarou:** supervision, writing – review and editing, investigation, writing – original draft, software, visualization, methodology, data curation. **Nikolaos Christodoulou:** methodology, supervision, writing – review and editing. **Andreas S. Lappas:** methodology, investigation, writing – review and editing, supervision, software, data curation. **Vasilios‐Panteleimon Bozikas:** supervision, writing – review and editing. **Myrto T. Samara:** conceptualization, methodology, supervision, writing – review and editing, investigation, project administration, validation.

## Conflicts of Interest

Anastasios Stefanou, Ioannis Anastasiou, Panagiota Fallon, Eleni Glarou, Andreas S. Lappas and Nikolaos Christodoulou have no conflicts of interest to disclose. Vasilios‐Panteleimon Bozikas has received honoraria as a consultant/advisor and/or for satellite symposiums from Johnson and Johnson, Viatris, Vian‐Vianex, Lundbeck, Innovis and Teva. Myrto T. Samara has received honoraria as a consultant/advisor and/or for lectures from Recordati, Lundbeck, and Viatris.

## Supporting information


**Data S1:** Supporting Information.

## Data Availability

The data that support the findings of this study are available from the corresponding author upon reasonable request.

## References

[jsr70231-bib-0001] Agnew, S. , A. Vallières , A. Hamilton , et al. 2021. “Adherence to Cognitive Behavior Therapy for Insomnia: An Updated Systematic Review.” Sleep Medicine Clinics 16, no. 1: 155–202. 10.1016/j.jsmc.2020.11.002. 33485527

[jsr70231-bib-0002] American Psychiatric Association . 2013. Diagnostic and Statistical Manual of Mental Disorders. 5th ed. American Psychiatric Association. 10.1176/appi.books.9780890425596.

[jsr70231-bib-0003] Arango, C. , J. K. Buitelaar , J. M. Fegert , et al. 2022. “Safety and Efficacy of Agomelatine in Children and Adolescents With Major Depressive Disorder Receiving Psychosocial Counselling: A Double‐Blind, Randomised, Controlled, Phase 3 Trial in Nine Countries.” Lancet Psychiatry 9, no. 2: 113–124. 10.1016/S2215-0366(21)00390-4. 34919834

[jsr70231-bib-0004] Astara, K. , A. Tsimpolis , K. Kalafatakis , et al. 2024. “Sleep Disorders and Alzheimer's Disease Pathophysiology: The Role of the Glymphatic System. A Scoping Review.” Mechanisms of Ageing and Development 217: 111899. 10.1016/j.mad.2023.111899. 38163471

[jsr70231-bib-0005] Avila, A. , X. Cardona , M. Martin‐Baranera , et al. 2015. “Agomelatine for Depression in Parkinson Disease: Additional Effect on Sleep and Motor Dysfunction.” Journal of Clinical Psychopharmacology 35, no. 6: 719–723. 10.1097/JCP.0000000000000404. 26444951

[jsr70231-bib-0006] Azadi, H. , P. Rashidpour , S. M. Yassini Ardekani , M. Nadi Sakhvidi , H. Afshang , and R. Bidaki . 2024. “The Effect of Adding Agomelatine to Escitalopram in the Treatment of Major Depressive Disorder.” Nevrologiya, Neiropsikhiatriya, Psikhosomatika = Neurology, Neuropsychiatry, Psychosomatics 16, no. 5: 24–29. 10.14412/2074-2711-2024-5-24-29.

[jsr70231-bib-0007] Ballester, P. , M. J. Martínez , M.‐M. Inda , et al. 2019. “Evaluation of Agomelatine for the Treatment of Sleep Problems in Adults With Autism Spectrum Disorder and Co‐Morbid Intellectual Disability.” Journal of Psychopharmacology 33, no. 11: 1395–1406. 10.1177/0269881119864968. 31423939

[jsr70231-bib-0008] Borestein, M. , L. V. Hedges , J. P. T. Higgins , and H. R. Rotherstein . 2009. Introduction to Meta‐Analysis. Wiley.

[jsr70231-bib-0009] Bourin, M. , and C. Prica . 2009. “Melatonin Receptor Agonist Agomelatine: A New Drug for Treating Unipolar Depression.” Current Pharmaceutical Design 15, no. 14: 1675–1682. 10.2174/138161209788168056. 19442180

[jsr70231-bib-0010] Bushnell, G. A. , T. Gerhard , K. Keyes , D. Hasin , M. Cerdá , and M. Olfson . 2022. “Association of Benzodiazepine Treatment for Sleep Disorders With Drug Overdose Risk Among Young People.” JAMA Network Open 5, no. 11: e2243215. 10.1001/jamanetworkopen.2022.43215. 36413369 PMC9682430

[jsr70231-bib-0011] Chattu, V. K. , M. D. Manzar , S. Kumary , D. Burman , D. W. Spence , and S. R. Pandi‐Perumal . 2019. “The Global Problem of Insufficient Sleep and Its Serious Public Health Implications.” Healthcare 7, no. 1: 1. 10.3390/healthcare7010001. PMC6473877 30577441

[jsr70231-bib-0012] Corruble, E. , C. de Bodinat , C. Belaïdi , G. M. Goodwin , and agomelatine study group . 2013. “Efficacy of Agomelatine and Escitalopram on Depression, Subjective Sleep and Emotional Experiences in Patients With Major Depressive Disorder: A 24‐Wk Randomized, Controlled, Double‐Blind Trial.” International Journal of Neuropsychopharmacology 16, no. 10: 2219–2234. 10.1017/S1461145713000679. 23823799

[jsr70231-bib-0013] Crescenzo, F. D. , G. L. D'Alò , E. G. Ostinelli , et al. 2022. “Comparative Effects of Pharmacological Interventions for the Acute and Long‐Term Management of Insomnia Disorder in Adults: A Systematic Review and Network Meta‐Analysis.” Lancet 400, no. 10347: 170–184. 10.1016/S0140-6736(22)00878-9. 35843245

[jsr70231-bib-0014] Dastan, F. , B. Gholizadeh Niari , P. Adimi Naghan , S. Barati , and R. Eskandari . 2023. “Evaluating the Effects of Agomelatine on Polysomnography Parameters in Patients With Obstructive Sleep Apnea.” Journal of Pharmacy & Pharmaceutical Sciences 25: 418–424. 10.18433/jpps33252. 36623475

[jsr70231-bib-0015] De Berardis, D. , M. Fornaro , N. Serroni , et al. 2015. “Agomelatine Beyond Borders: Current Evidences of Its Efficacy in Disorders Other Than Major Depression.” International Journal of Molecular Sciences 16, no. 1: 1111–1130. 10.3390/ijms16011111. 25569089 PMC4307293

[jsr70231-bib-0016] Deeks, J. J. , J. P. T. Higgins , D. G. Altman , J. E. McKenzie , and A. A. Veroniki . 2024. “Chapter 10: Analysing Data and Undertaking Meta‐Analyses.” In Cochrane Handbook for Systematic Reviews of Interventions (6.5), edited by J. P. T. Higgins , J. Thomas , J. Chandler , M. Cumpston , T. Li , M. J. Page , and V. A. Welch . Cochrane.

[jsr70231-bib-0017] Dettori, J. R. , D. C. Norvell , and J. R. Chapman . 2022. “Fixed‐Effect vs Random‐Effects Models for Meta‐Analysis: 3 Points to Consider.” Global Spine Journal 12, no. 7: 1624–1626. 10.1177/21925682221110527. 35723546 PMC9393987

[jsr70231-bib-0018] Duval, S. , and R. Tweedie . 2000. “Trim and Fill: A Simple Funnel‐Plot‐Based Method of Testing and Adjusting for Publication Bias in Meta‐Analysis.” Biometrics 56, no. 2: 455–463. 10.1111/j.0006-341x.2000.00455.x. 10877304

[jsr70231-bib-0019] Egger, M. , G. Davey Smith , M. Schneider , and C. Minder . 1997. “Bias in Meta‐Analysis Detected by a Simple, Graphical Test.” BMJ 315, no. 7109: 629–634. 10.1136/bmj.315.7109.629. 9310563 PMC2127453

[jsr70231-bib-0020] Fabbri, M. , A. Beracci , M. Martoni , D. Meneo , L. Tonetti , and V. Natale . 2021. “Measuring Subjective Sleep Quality: A Review.” International Journal of Environmental Research and Public Health 18, no. 3: 3. 10.3390/ijerph18031082. PMC7908437 33530453

[jsr70231-bib-0021] Fang, H. , S. Tu , J. Sheng , and A. Shao . 2019. “Depression in Sleep Disturbance: A Review on a Bidirectional Relationship, Mechanisms and Treatment.” Journal of Cellular and Molecular Medicine 23, no. 4: 2324–2332. 10.1111/jcmm.14170. 30734486 PMC6433686

[jsr70231-bib-0022] Furukawa, T. A. , C. Barbui , A. Cipriani , P. Brambilla , and N. Watanabe . 2006. “Imputing Missing Standard Deviations in Meta‐Analyses Can Provide Accurate Results.” Journal of Clinical Epidemiology 59, no. 1: 7–10. 10.1016/j.jclinepi.2005.06.006. 16360555

[jsr70231-bib-0023] Furukawa, T. A. , G. Salanti , L. Z. Atkinson , et al. 2016. “Comparative Efficacy and Acceptability of First‐Generation and Second‐Generation Antidepressants in the Acute Treatment of Major Depression: Protocol for a Network Meta‐Analysis.” BMJ Open 6, no. 7: e010919. 10.1136/bmjopen-2015-010919. PMC4947714 27401359

[jsr70231-bib-0024] Geiger‐Brown, J. M. , V. E. Rogers , W. Liu , E. M. Ludeman , K. D. Downton , and M. Diaz‐Abad . 2015. “Cognitive Behavioral Therapy in Persons With Comorbid Insomnia: A Meta‐Analysis.” Sleep Medicine Reviews 23: 54–67. 10.1016/j.smrv.2014.11.007. 25645130

[jsr70231-bib-0025] Gottlieb, D. J. , N. M. Punjabi , A. B. Newman , et al. 2005. “Association of Sleep Time With Diabetes Mellitus and Impaired Glucose Tolerance.” Archives of Internal Medicine 165, no. 8: 863–867. 10.1001/archinte.165.8.863. 15851636

[jsr70231-bib-0026] Guaiana, G. , S. Gupta , D. Chiodo , S. J. Davies , K. Haederle , and M. Koesters . 2013. “Agomelatine Versus Other Antidepressive Agents for Major Depression.” Cochrane Database of Systematic Reviews 2013, no. 12: CD008851. 10.1002/14651858.CD008851.pub2. 24343836 PMC11289707

[jsr70231-bib-0027] Gunja, N. 2013. “In the Zzz Zone: The Effects of Z‐Drugs on Human Performance and Driving.” Journal of Medical Toxicology 9, no. 2: 163–171. 10.1007/s13181-013-0294-y. 23456542 PMC3657033

[jsr70231-bib-0028] Guo, Y.‐H. , L. Zhou , Z.‐A. Cui , et al. 2023. “Efficacy and Safety of Agomelatine in the Treatment of Patients With Depressive Disorder: A Meta‐Analysis.” Medicine 102, no. 45: e35871. 10.1097/MD.0000000000035871. 37960759 PMC10637518

[jsr70231-bib-0029] Heun, R. , A. Ahokas , P. Boyer , et al. 2013. “The Efficacy of Agomelatine in Elderly Patients With Recurrent Major Depressive Disorder: A Placebo‐Controlled Study.” Journal of Clinical Psychiatry 74, no. 6: 587–594. 10.4088/JCP.12m08250. 23842010

[jsr70231-bib-0030] Higgins, J. P. T. , D. G. Altman , P. C. Gøtzsche , et al. 2011. “The Cochrane Collaboration's Tool for Assessing Risk of Bias in Randomised Trials.” BMJ 343: d5928. 10.1136/bmj.d5928. 22008217 PMC3196245

[jsr70231-bib-0031] Higgins, J. P. T. , and S. Green . 2011. Cochrane Handbook for Systematic Reviews of Interventions. Version 5.1.0. Cochrane Collaboration. https://handbook‐5‐1.cochrane.org/.

[jsr70231-bib-0032] Higgins, J. P. T. , T. Li , and J. J. Deeks . 2023. “Choosing Effect Measures and Computing Estimates of Effect.” In Cochrane Handbook for Systematic Reviews of Interventions, edited by J. P. T. Higgins , J. Thomas , J. Chandler , M. Cumpston , T. Li , M. J. Page , and V. A. Welch . Cochrane. https://www.cochrane.org/authors/handbooks‐and‐manuals/handbook/current.

[jsr70231-bib-0033] Jung, M. E. , D. B. Metzger , and J. Hall . 2020. “The Long‐Term but Not Short‐Term Use of Benzodiazepine Impairs Motoric Function and Upregulates Amyloid β in Part Through the Suppression of Translocator Protein.” Pharmacology Biochemistry and Behavior 191: 172873. 10.1016/j.pbb.2020.172873. 32105662

[jsr70231-bib-0034] Kasper, S. , G. Hajak , K. Wulff , et al. 2010. “Efficacy of the Novel Antidepressant Agomelatine on the Circadian Rest‐Activity Cycle and Depressive and Anxiety Symptoms in Patients With Major Depressive Disorder: A Randomized, Double‐Blind Comparison With Sertraline.” Journal of Clinical Psychiatry 71, no. 2: 109–120. 10.4088/JCP.09m05347blu. 20193645

[jsr70231-bib-0035] Katz, D. A. , and C. A. McHorney . 2002. “The Relationship Between Insomnia and Health‐Related Quality of Life in Patients With Chronic Illness.” Journal of Family Practice 51, no. 3: 229–235. 11978233

[jsr70231-bib-0036] Kennedy, S. H. , A. Avedisova , N. Giménez‐Montesinos , C. Belaïdi , C. de Bodinat , and Agomelatine Study Group . 2014. “A Placebo‐Controlled Study of Three Agomelatine Dose Regimens (10 Mg, 25 Mg, 25‐50 Mg) in Patients With Major Depressive Disorder.” European Neuropsychopharmacology: The Journal of the European College of Neuropsychopharmacology 24, no. 4: 553–563. 10.1016/j.euroneuro.2014.01.006. 24530273

[jsr70231-bib-0037] Kennedy, S. H. , and R. Emsley . 2006. “Placebo‐Controlled Trial of Agomelatine in the Treatment of Major Depressive Disorder.” European Neuropsychopharmacology: The Journal of the European College of Neuropsychopharmacology 16, no. 2: 93–100. 10.1016/j.euroneuro.2005.09.002. 16249073

[jsr70231-bib-0038] Koesters, M. , G. Guaiana , A. Cipriani , T. Becker , and C. Barbui . 2013. “Agomelatine Efficacy and Acceptability Revisited: Systematic Review and Meta‐Analysis of Published and Unpublished Randomised Trials.” British Journal of Psychiatry: The Journal of Mental Science 203, no. 3: 179–187. 10.1192/bjp.bp.112.120196. 23999482

[jsr70231-bib-0039] Kokkali, M. , E. Pinioti , A. S. Lappas , N. Christodoulou , and M. T. Samara . 2024. “Effects of Trazodone on Sleep: A Systematic Review and Meta‐Analysis.” CNS Drugs 38, no. 10: 753–769. 10.1007/s40263-024-01110-2. 39123094

[jsr70231-bib-0040] Krystal, A. D. 2012. “Psychiatric Disorders and Sleep.” Neurologic Clinics 30, no. 4: 1389–1413. 10.1016/j.ncl.2012.08.018. 23099143 PMC3493205

[jsr70231-bib-0041] Lappas, A. S. , E. Glarou , Z. A. Polyzopoulou , et al. 2024. “Pharmacotherapy for Sleep Disturbances in Post‐Traumatic Stress Disorder (PTSD): A Network Meta‐Analysis.” Sleep Medicine 119: 467–479. 10.1016/j.sleep.2024.05.032. 38795401

[jsr70231-bib-0042] Lappas, A. S. , Z. A. Polyzopoulou , N. Christodoulou , V.‐P. Bozikas , and M. T. Samara . 2024. “Effects of Antidepressants on Sleep in Post‐Traumatic Stress Disorder: An Overview of Reviews.” Current Neuropharmacology 22, no. 4: 749–805. 10.2174/1570159X21666230801144328. 37533247 PMC10845105

[jsr70231-bib-0043] Lawson, L. P. , A. L. Richdale , K. Denney , and E. M. J. Morris . 2023. “ACT‐i, an Insomnia Intervention for Autistic Adults: A Pilot Study.” Behavioural and Cognitive Psychotherapy 51, no. 2: 146–163. 10.1017/S1352465822000571. 36537291

[jsr70231-bib-0044] Léger, D. , C. Guilleminault , G. Bader , E. Lévy , and M. Paillard . 2002. “Medical and Socio‐Professional Impact of Insomnia.” Sleep 25, no. 6: 621–625. 10.1093/sleep/25.6.621. 12224841

[jsr70231-bib-0045] Lemoine, P. , C. Guilleminault , and E. Alvarez . 2007. “Improvement in Subjective Sleep in Major Depressive Disorder With a Novel Antidepressant, Agomelatine: Randomized, Double‐Blind Comparison With Venlafaxine.” Journal of Clinical Psychiatry 68, no. 11: 1723–1732. 10.4088/jcp.v68n1112. 18052566

[jsr70231-bib-0046] Lemola, S. , T. Ledermann , and E. M. Friedman . 2013. “Variability of Sleep Duration Is Related to Subjective Sleep Quality and Subjective Well‐Being: An Actigraphy Study.” PLoS One 8, no. 8: e71292. 10.1371/journal.pone.0071292. 23967186 PMC3743871

[jsr70231-bib-0047] Leproult, R. , A. Van Onderbergen , M. L'Hermite‐Balériaux , E. Van Cauter , and G. Copinschi . 2005. “Phase‐Shifts of 24‐h Rhythms of Hormonal Release and Body Temperature Following Early Evening Administration of the Melatonin Agonist Agomelatine in Healthy Older Men.” Clinical Endocrinology 63, no. 3: 298–304. 10.1111/j.1365-2265.2005.02341.x. 16117817

[jsr70231-bib-0048] Liu, J. , S. J. Clough , A. J. Hutchinson , et al. 2016. “MT1 and MT2 Melatonin Receptors: A Therapeutic Perspective.” Annual Review of Pharmacology and Toxicology 56: 361–383. 10.1146/annurev-pharmtox-010814-124742. PMC5091650 26514204

[jsr70231-bib-0049] Lôo, H. , A. Hale , and H. D'haenen . 2002. “Determination of the Dose of Agomelatine, a Melatoninergic Agonist and Selective 5‐HT2C Antagonist, in the Treatment of Major Depressive Disorder: A Placebo‐Controlled Dose Range Study.” International Clinical Psychopharmacology 17, no. 5: 239. 12177586 10.1097/00004850-200209000-00004

[jsr70231-bib-0050] Lucchetta, R. C. , B. P. M. da Mata , and P. d. C. Mastroianni . 2018. “Association Between Development of Dementia and Use of Benzodiazepines: A Systematic Review and Meta‐Analysis.” Pharmacotherapy: The Journal of Human Pharmacology and Drug Therapy 38, no. 10: 1010–1020. 10.1002/phar.2170. 30098211

[jsr70231-bib-0051] Mahdavi, S. M. , B. Shariati , M. Shalbafan , et al. 2022. “The Effectiveness of Pregabalin With or Without Agomelatine in the Treatment of Chronic Low Back Pain: A Double‐Blind, Placebo‐Controlled, Randomized Clinical Trial.” BMC Pharmacology and Toxicology 23, no. 1: 70. 10.1186/s40360-022-00612-3. 36104745 PMC9476640

[jsr70231-bib-0052] Marey, C. , and Servier Laboratories . 2020. “Evaluation of Efficacy and Clinical Benefit of Agomelatine in Patients With Major Depressive Disorder Compared to Serotonin‐Norepinephrine Reuptake Inhibitor (SNRI).” ISRCTN Registry. 10.1186/ISRCTN96725312.

[jsr70231-bib-0053] Marović, I. , I. Marinović , V. Bačić Vrca , and I. Samardžić . 2024. “Assessment of Potential Drug–Drug Interactions of Psycholeptics and Antidepressants in Outpatient Settings.” Pharmacy 12, no. 6: 6. 10.3390/pharmacy12060174. PMC11587429 39585100

[jsr70231-bib-0054] Medic, G. , M. Wille , and M. E. Hemels . 2017. “Short‐ and Long‐Term Health Consequences of Sleep Disruption.” Nature and Science of Sleep 9: 151–161. 10.2147/NSS.S134864. PMC5449130 28579842

[jsr70231-bib-0055] Meier‐Ewert, H. K. , P. M. Ridker , N. Rifai , et al. 2004. “Effect of Sleep Loss on C‐Reactive Protein, an Inflammatory Marker of Cardiovascular Risk.” Journal of the American College of Cardiology 43, no. 4: 678–683. 10.1016/j.jacc.2003.07.050. 14975482

[jsr70231-bib-0056] Mi, W.‐F. , S. Tabarak , L. Wang , et al. 2020. “Effects of Agomelatine and Mirtazapine on Sleep Disturbances in Major Depressive Disorder: Evidence From Polysomnographic and Resting‐State Functional Connectivity Analyses.” Sleep 43, no. 11: zsaa092. 10.1093/sleep/zsaa092. 32406918

[jsr70231-bib-0057] Millan, M. J. 2022. “Agomelatine for the Treatment of Generalized Anxiety Disorder: Focus on Its Distinctive Mechanism of Action.” Therapeutic Advances in Psychopharmacology 12: 20451253221105128. 10.1177/20451253221105128. 35795687 PMC9251978

[jsr70231-bib-0058] Moher, D. , A. Liberati , J. Tetzlaff , D. G. Altman , and Group, T. P . 2009. “Preferred Reporting Items for Systematic Reviews and Meta‐Analyses: The PRISMA Statement.” PLoS Medicine 6, no. 7: e1000097. 10.1371/journal.pmed.1000097. 19621072 PMC2707599

[jsr70231-bib-0059] Montejo, A. L. , J. Deakin , R. Gaillard , et al. 2015. “Better Sexual Acceptability of Agomelatine (25 and 50 Mg) Compared to Escitalopram (20 Mg) in Healthy Volunteers. A 9‐Week, Placebo‐Controlled Study Using the PRSexDQ Scale.” Journal of Psychopharmacology 29, no. 10: 1119–1128. 10.1177/0269881115599385. 26268533

[jsr70231-bib-0060] Nejati, A. , A. Bazrafshan , and S. H. Mosavat . 2024. “Agomelatine Efficacy in Treatment Resistant Obsessive‐Compulsive Disorder: A Randomized Controlled Trial.” International Journal of Psychiatry in Medicine 59, no. 5: 545–555. 10.1177/00912174231225763. 38156645

[jsr70231-bib-0061] Nijhof, D. , C. Melville , E. Rydzewska , G. Pavlopoulou , L. Meehan , and M. Gardani . 2024. “Experiences of and Treatment Preferences for Insomnia in Autistic Adults: An Interpretative Phenomenological Analysis.” Sleep Medicine 122: 163–170. 10.1016/j.sleep.2024.08.011. 39178754

[jsr70231-bib-0062] Novartis Pharmaceuticals . 2020. A 8‐week, Randomized, Double‐Blind, Placebo‐controlled, Parallel‐group, Multi‐center Study of the Efficacy and Safety of Agomelatine 0.5 mg and 1 mg Sublingual Tablets Administered Once Daily in Patients With Major Depressive Disorder (MDD) (Clinical Trial Registration CAGO178C2302) Novartis Pharmaceuticals. https://clinicaltrials.gov/study/NCT01110902.

[jsr70231-bib-0063] Olié, J. P. , and S. Kasper . 2007. “Efficacy of Agomelatine, a MT1/MT2 Receptor Agonist With 5‐HT2C Antagonistic Properties, in Major Depressive Disorder.” International Journal of Neuropsychopharmacology 10, no. 5: 661–673. 10.1017/S1461145707007766. 17477888

[jsr70231-bib-0064] Ouzzani, M. , H. Hammady , Z. Fedorowicz , and A. Elmagarmid . 2016. “Rayyan—A Web and Mobile App for Systematic Reviews.” Systematic Reviews 5, no. 1: 210. 10.1186/s13643-016-0384-4. 27919275 PMC5139140

[jsr70231-bib-0065] Page, M. J. , J. E. McKenzie , P. M. Bossuyt , et al. 2021. “The PRISMA 2020 Statement: An Updated Guideline for Reporting Systematic Reviews.” BMJ 372: n71. 10.1136/bmj.n71. 33782057 PMC8005924

[jsr70231-bib-0066] Pandi‐Perumal, S. R. , A. Moscovitch , V. Srinivasan , D. W. Spence , D. P. Cardinali , and G. M. Brown . 2009. “Bidirectional Communication Between Sleep and Circadian Rhythms and Its Implications for Depression: Lessons From Agomelatine.” Progress in Neurobiology 88, no. 4: 264–271. 10.1016/j.pneurobio.2009.04.007. 19454302

[jsr70231-bib-0067] Poluéktov, M. G. , and Y. I. Levin . 2013. “The Results of the Russian Multicenter Open Observational Non‐Comparative Study on the Efficacy and Safety of Valdoxan (Agomelatin) in the Treatment of Patients With Major Depressive Disorder and Insomnia (The VIVALDI Study).” Zhurnal Nevrologii i Psikhiatrii Imeni S.S. Korsakova 113, no. 12: 39–44. 24430033

[jsr70231-bib-0068] Porteous, M. , S. Fogel , L. Ray , et al. 2021. “Increased Spindle Density Correlates With Sleep Continuity Improvements Following an Eight‐Week Course of a Melatonin Agonist in People With Depression: A Proof‐Of‐Concept Study With Agomelatine.” European Journal of Neuroscience 54, no. 3: 5112–5119. 10.1111/ejn.15340. 34089546

[jsr70231-bib-0069] Quera Salva, M.‐A. , B. Vanier , J. Laredo , et al. 2007. “Major Depressive Disorder, Sleep EEG and Agomelatine: An Open‐Label Study.” International Journal of Neuropsychopharmacology 10, no. 5: 691–696. 10.1017/S1461145707007754. 17477886

[jsr70231-bib-0070] Quera‐Salva, M.‐A. , G. Hajak , P. Philip , et al. 2011. “Comparison of Agomelatine and Escitalopram on Nighttime Sleep and Daytime Condition and Efficacy in Major Depressive Disorder Patients.” International Clinical Psychopharmacology 26, no. 5: 252–262. 10.1097/YIC.0b013e328349b117. 21829106

[jsr70231-bib-0071] R Core Team . 2024. R: A Language and Environment for Statistical Computing. (Version 4.4.1) [Computer Software]. R Foundation for Statistical Computing. https://www.R‐project.org/.

[jsr70231-bib-0072] Rascati, K. 1995. “Drug Utilization Review of Concomitant Use of Specific Serotonin Reuptake Inhibitors or Clomipramine With Antianxiety/Sleep Medications.” Clinical Therapeutics 17, no. 4: 786–790. 10.1016/0149-2918(95)80055-7. 8565041

[jsr70231-bib-0073] Riemann, D. , C. Baglioni , C. Bassetti , et al. 2017. “European Guideline for the Diagnosis and Treatment of Insomnia.” Journal of Sleep Research 26, no. 6: 675–700. 10.1111/jsr.12594. 28875581

[jsr70231-bib-0074] Rouillon, F. , and Servier Laboratories . 2008. Efficacy and Safety of Two Doses of S 90098 (1 and 2 mg/day), Sublingual Formulation for 8 weeks in Out‐patients with Major depressive disorder: An 8‐week randomised, double‐Blind, Fixed Dose, International, Multicentre, Placebo‐Controlled Study With Parallel Groups, Followed by an Extension Double‐Blind Treatment Period for 16 weeks (Controlled‐Trials.Com [ISRCTN38378163; CL2‐90098‐005]). www.controlled‐trials.com.

[jsr70231-bib-0075] Salin, K. , N. Kasitanon , B. Maneeton , and W. Louthrenoo . 2019. The Effect of Agomelatine on Sleep Disturbance, Depression, and Anxiety in Patients with Systemic Lupus Erythematosus: A Randomized, DoubleBlinded Placebo‐Controlled Trial. The 35th Annual Meeting the Royal College of Physicians of Thailand “Towards Better and Safer Patient Care,” Chonburi, PEACH Royal Cliff Beach Resort, Pattaya, Thailand, 2019, April 25.

[jsr70231-bib-0076] Samara, M. T. 2022. “What Is the Right Drug for Insomnia Disorder?” Lancet 400, no. 10347: 139–141. 10.1016/S0140-6736(22)01322-8. 35843230

[jsr70231-bib-0077] Samara, M. T. , M. Huhn , V. Chiocchia , et al. 2020. “Efficacy, Acceptability, and Tolerability of All Available Treatments for Insomnia in the Elderly: A Systematic Review and Network Meta‐Analysis.” Acta Psychiatrica Scandinavica 142, no. 1: 6–17. 10.1111/acps.13201. 32521042

[jsr70231-bib-0078] Sateia, M. J. 2014. “International Classification of Sleep Disorders‐Third Edition.” Chest 146, no. 5: 1387–1394. 10.1378/chest.14-0970. 25367475

[jsr70231-bib-0079] Sateia, M. J. , D. J. Buysse , A. D. Krystal , D. N. Neubauer , and J. L. Heald . 2017. “Clinical Practice Guideline for the Pharmacologic Treatment of Chronic Insomnia in Adults: An American Academy of Sleep Medicine Clinical Practice Guideline.” Journal of Clinical Sleep Medicine 13, no. 2: 307–349. 10.5664/jcsm.6470. 27998379 PMC5263087

[jsr70231-bib-0080] Scharner, V. , L. Hasieber , A. Sönnichsen , and E. Mann . 2022. “Efficacy and Safety of Z‐Substances in the Management of Insomnia in Older Adults: A Systematic Review for the Development of Recommendations to Reduce Potentially Inappropriate Prescribing.” BMC Geriatrics 22, no. 1: 87. 10.1186/s12877-022-02757-6. 35100976 PMC9887772

[jsr70231-bib-0081] Sejbuk, M. , I. Mirończuk‐Chodakowska , and A. M. Witkowska . 2022. “Sleep Quality: A Narrative Review on Nutrition, Stimulants, and Physical Activity as Important Factors.” Nutrients 14, no. 9: 9. 10.3390/nu14091912. PMC9103473 35565879

[jsr70231-bib-0082] Servier Laboratories . 2009a. Efficacy and Safety of 3 doses (0.25, 0.5 and 1mg/day) of agomelatine sublingual administration over an 8‐week treatment period, in out‐patients with Major Depressive Disorder. An 8‐week randomised, double‐blind, fixed dose, international multicentre, placebo‐controlled study with parallel groups, followed by an extension double‐blind treatment period of 16 weeks. ([CL2‐90098‐009; EUCTR2009‐014045‐92]. EU Clinical Trials Register). www.clinicaltrialsregister.eu.

[jsr70231-bib-0083] Servier Laboratories . 2009b. Valdoxan 25 mg Film‐coated tablets. Summary of Product Characteristics. European Medicines Agency.

[jsr70231-bib-0084] Shokrani, M. , S. Askari , N. Eissazade , et al. 2023. “Agomelatine Augmentation of Sertraline in the Treatment of Moderate to Severe Obsessive‐Compulsive Disorder: A Randomized Double‐Blinded Placebo‐Controlled Clinical Trial.” BMC Psychiatry 23, no. 1: 686. 10.1186/s12888-023-05189-7. 37735631 PMC10512611

[jsr70231-bib-0085] Shu, L. , A. H. Sulaiman , Y. S. Huang , C. Fones Soon Leng , V. S. Crutel , and Y. S. Kim . 2014. “Comparable Efficacy and Safety of 8 Weeks Treatment With Agomelatine 25‐50mg or Fluoxetine 20‐40mg in Asian Out‐Patients With Major Depressive Disorder.” Asian Journal of Psychiatry 8: 26–32. 10.1016/j.ajp.2013.09.009. 24655622

[jsr70231-bib-0086] Stahl, S. 2021a. Stahl's Essential Psychopharmacology. Neuroscientific Basis and Practical Applications. 5th ed. Cambridge University Press.

[jsr70231-bib-0087] Stahl, S. 2021b. Stahl's Essential Psychopharmacology. Prescriber's Guide. 7th ed. Cambridge University Press.

[jsr70231-bib-0088] Stahl, S. , M. Fava , M. H. Trivedi , A. Caputo , A. Shah , and A. Post . 2010. “Agomelatine in the Treatment of Major Depressive Disorder: An 8‐Week, Multicenter, Randomized, Placebo‐Controlled Trial.” Journal of Clinical Psychiatry 71, no. 5: 669. 10.4088/JCP.09m05471blu. 20361916

[jsr70231-bib-0089] Stein, D. J. , A. Ahokas , C. Albarran , V. Olivier , and C. Allgulander . 2012. “Agomelatine Prevents Relapse in Generalized Anxiety Disorder: A 6‐Month Randomized, Double‐Blind, Placebo‐Controlled Discontinuation Study.” Journal of Clinical Psychiatry 73, no. 7: 665. 10.4088/JCP.11m07493. 22901350

[jsr70231-bib-0090] Stein, D. J. , A. Ahokas , M. Jarema , et al. 2017. “Efficacy and Safety of Agomelatine (10 or 25 Mg/Day) in Non‐Depressed Out‐Patients With Generalized Anxiety Disorder: A 12‐Week, Double‐Blind, Placebo‐Controlled Study.” European Neuropsychopharmacology 27, no. 5: 526–537. 10.1016/j.euroneuro.2017.02.007. 28298261

[jsr70231-bib-0091] Stein, D. J. , A. Ahokas , M. S. Márquez , et al. 2014. “Agomelatine in Generalized Anxiety Disorder: An Active Comparator and Placebo‐Controlled Study.” Journal of Clinical Psychiatry 75, no. 4: 663. 10.4088/JCP.13m08433. 24569045

[jsr70231-bib-0092] Stein, D. J. , A. A. Ahokas , and C. de Bodinat . 2008. “Efficacy of Agomelatine in Generalized Anxiety Disorder: A Randomized, Double‐Blind, Placebo‐Controlled Study.” Journal of Clinical Psychopharmacology 28, no. 5: 561–566. 10.1097/JCP.0b013e318184ff5b. 18794654

[jsr70231-bib-0093] Stein, D. J. , J.‐P. Khoo , A. Ahokas , et al. 2018. “12‐Week Double‐Blind Randomized Multicenter Study of Efficacy and Safety of Agomelatine (25–50 Mg/Day) *Versus* Escitalopram (10–20 Mg/Day) in Out‐Patients With Severe Generalized Anxiety Disorder.” European Neuropsychopharmacology 28, no. 8: 970–979. 10.1016/j.euroneuro.2018.05.006. 30135032

[jsr70231-bib-0094] Streatfeild, J. , J. Smith , D. Mansfield , L. Pezzullo , and D. Hillman . 2021. “The Social and Economic Cost of Sleep Disorders.” Sleep 44, no. 11: zsab132. 10.1093/sleep/zsab132. 34015136

[jsr70231-bib-0095] Su, Q. , T. Li , G.‐W. Liu , et al. 2023. “Agomelatine: A Potential Novel Approach for the Treatment of Memory Disorder in Neurodegenerative Disease.” Neural Regeneration Research 18, no. 4: 727–733. 10.4103/1673-5374.353479. 36204828 PMC9700086

[jsr70231-bib-0096] Taylor, D. , A. Sparshatt , S. Varma , and O. Olofinjana . 2014. “Antidepressant Efficacy of Agomelatine: Meta‐Analysis of Published and Unpublished Studies.” BMJ 348: g1888. 10.1136/bmj.g1888. 24647162 PMC3959623

[jsr70231-bib-0097] Tchekalarova, J. , L. Kortenska , N. Ivanova , M. Atanasova , and P. Marinov . 2020. “Agomelatine Treatment Corrects Impaired Sleep‐Wake Cycle and Sleep Architecture and Increases MT1 Receptor as Well as BDNF Expression in the Hippocampus During the Subjective Light Phase of Rats Exposed to Chronic Constant Light.” Psychopharmacology 237, no. 2: 503–518. 10.1007/s00213-019-05385-y. 31720718

[jsr70231-bib-0098] Thase, M. E. 2006. “Pharmacotherapy of Bipolar Depression: An Update.” Current Psychiatry Reports 8, no. 6: 478–488. 10.1007/s11920-006-0055-6. 17094928

[jsr70231-bib-0099] Thorpy, M. 2017. “International Classification of Sleep Disorders.” In Sleep Disorders Medicine: Basic Science, Technical Considerations and Clinical Aspects, edited by S. Chokroverty , 475–484. Springer. 10.1007/978-1-4939-6578-6_27.

[jsr70231-bib-0100] Trikalinos, T. A. , R. Churchill , M. Ferri , et al. 2004. “Effect Sizes in Cumulative Meta‐Analyses of Mental Health Randomized Trials Evolved Over Time.” Journal of Clinical Epidemiology 57, no. 11: 1124–1130. 10.1016/j.jclinepi.2004.02.018. 15612138

[jsr70231-bib-0101] Tsuno, N. , A. Besset , and K. Ritchie . 2005. “Sleep and Depression.” Journal of Clinical Psychiatry 66, no. 10: 19685. 10.4088/jcp.v66n1008 16259539

[jsr70231-bib-0102] Wang, C. , and D. M. Holtzman . 2020. “Bidirectional Relationship Between Sleep and Alzheimer's Disease: Role of Amyloid, Tau, and Other Factors.” Neuropsychopharmacology 45, no. 1: 104–120. 10.1038/s41386-019-0478-5. 31408876 PMC6879647

[jsr70231-bib-0103] World Health Organization . 2019. International Classification of Diseases, Eleventh Revision (ICD‐ 11). https://icd.who.int/browse11.

[jsr70231-bib-0104] Yatham, L. N. , E. Vieta , G. M. Goodwin , et al. 2016. “Agomelatine or Placebo as Adjunctive Therapy to a Mood Stabiliser in Bipolar I Depression: Randomised Double‐Blind Placebo‐Controlled Trial.” British Journal of Psychiatry 208, no. 1: 78–86. 10.1192/bjp.bp.114.147587. 25999335

[jsr70231-bib-0105] Zajecka, J. , A. Schatzberg , S. Stahl , A. Shah , A. Caputo , and A. Post . 2010. “Efficacy and Safety of Agomelatine in the Treatment of Major Depressive Disorder: A Multicenter, Randomized, Double‐Blind, Placebo‐Controlled Trial.” Journal of Clinical Psychopharmacology 30, no. 2: 135–144. 10.1097/JCP.0b013e3181d420a7. 20520286

[jsr70231-bib-0106] Zhou, F.‐C. , Y. Yang , Y.‐Y. Wang , et al. 2020. “Cognitive Behavioural Therapy for Insomnia Monotherapy in Patients With Medical or Psychiatric Comorbidities: A Meta‐Analysis of Randomized Controlled Trials.” Psychiatric Quarterly 91, no. 4: 1209–1224. 10.1007/s11126-020-09820-8. 32860556

[jsr70231-bib-0107] Zielinski, M. R. , J. T. McKenna , R. W. McCarley , M. R. Zielinski , J. T. McKenna , and R. W. McCarley . 2016. “Functions and Mechanisms of Sleep.” AIMS Neuroscience 3, no. 1: 67–104. 10.3934/Neuroscience.2016.1.67. 28413828 PMC5390528

[jsr70231-bib-0108] Zohar, & Servier Laboratories . 2009. Efficacy of Agomelatine 25 mg/day (With Possible Increase to 50 mg/day After 8 Weeks of treatment) given orally during 16 Weeks in Patients with Obsessive‐Compulsive Disorder. A randomised, Double‐Blind, Placebo‐Controlled, Parallel Groups, International Study. ([CL2‐20098‐072;EUCTR2009‐016713‐20]. EU Clinical Trials Register). www.clinicaltrialsregister.eu.

[jsr70231-bib-0109] Zupancic, M. , and C. Guilleminault . 2006. “Agomelatine.” CNS Drugs 20, no. 12: 981–992. 10.2165/00023210-200620120-00003. 17140278

