# Supplementary material for: Effects of Agomelatine on Sleep Across Populations: A Systematic Review and Meta‐Analysis
Source: J Sleep Res. 2025 Nov 2;35(3):e70231. doi: 10.1111/jsr.70231 (PMC13193371; doi:10.1111/jsr.70231)
Supplement: Supplementary file 1 — Data S1: Supporting Information. [file JSR-35-e70231-s001.docx]

**Supplementary Material**

**Table of Contents**

[1. **PRISMA Checklist** 3](#_Toc191285952)

[2. **Protocol of the systematic review and meta-analysis** 6](#_Toc191285953)

[**Effects of Agomelatine on sleep: a systematic review and meta-analysis** 6](#_Toc191285954)

[**Review Question** 6](#_Toc191285955)

[**Types of Study to be Included** 7](#_Toc191285956)

[**Condition or Domain Being Studied** 7](#_Toc191285957)

[**Participants/Population** 7](#_Toc191285958)

[**Intervention(s), Exposure(s)** 7](#_Toc191285959)

[**Comparator(s)/Control** 7](#_Toc191285960)

[**Main Outcome(s)** 8](#_Toc191285961)

[**Measures of Effect** 8](#_Toc191285962)

[**Additional Outcome(s)** 8](#_Toc191285963)

[**Measures of Effect** 9](#_Toc191285964)

[**Data Extraction (Selection and Coding)** 10](#_Toc191285965)

[**Risk of Bias (Quality) Assessment** 10](#_Toc191285966)

[**Strategy for Data Synthesis** 10](#_Toc191285967)

[**Analysis of Subgroups or Subsets** 11](#_Toc191285968)

[**Sensitivity Analyses:** 11](#_Toc191285969)

[**Contact Details for Further Information** 12](#_Toc191285970)

[**Organisational Affiliation of The Review** 12](#_Toc191285971)

[**Review Team Members and Their Organisational Affiliations [1 change]** 12](#_Toc191285972)

[**Type and Method of Review** 12](#_Toc191285973)

[3. **Deviations from the protocol.** 13](#_Toc191285974)

[4. **Search strategy** 14](#_Toc191285975)

[5.  **PRISMA Flow Diagram** 15](#_Toc191285976)

[6. **Table of Included Studies** 16](#_Toc191285977)

[7. **Risk of bias** 29](#_Toc191285978)

[8. **Ongoing Studies** 31](#_Toc191285979)

[9. **Forest plots for all outcomes** 37](#_Toc191285980)

[**9.1.** **Primary Outcomes** 37](#_Toc191285981)

[**9.1.1.** **Total Sleep Time (measured in minutes)** 37](#_Toc191285982)

[**9.1.2.** **Quality of Sleep (measured by PSQI and LSEQ)** 38](#_Toc191285983)

[**9.1.3.** **Insomnia as treatment emergent adverse effect.** 41](#_Toc191285984)

[**9.1.4.** **Somnolence as treatment emergent adverse effect** 43](#_Toc191285985)

[**9.2.** **Secondary Outcomes** 47](#_Toc191285986)

[**9.2.1.** **Sleep onset latency (Getting to sleep score measured in LSEQ)** 47](#_Toc191285987)

[**9.2.2.** **Sleep onset latency (minutes)** 47](#_Toc191285988)

[**9.2.3.** **Number of nocturnal awakenings** 48](#_Toc191285989)

[**9.2.4.** **Nocturnal time spent awake after sleep onset** 48](#_Toc191285990)

[**9.2.5.** **Daytime impairment (Sleep awakening score)** 49](#_Toc191285991)

[**9.2.6.** **Number of dropouts due to adverse effects** 49](#_Toc191285992)

[**9.2.7.** **Number of dropouts due to sleep-related adverse effects** 50](#_Toc191285993)

[**9.2.8.** **Number of participants with adverse effects** 50](#_Toc191285994)

[**9.2.9.** **Number of participants with sleep related adverse effects** 51](#_Toc191285995)

[**9.2.10.** **Other (Behaviour Integrity)** 51](#_Toc191285996)

[10. **Assessment of Publication bias** 52](#_Toc191285997)

[**10.1.** **Funnel plots: Primary outcomes with at least 10 studies** 52](#_Toc191285998)

[**10.1.1.** **Funnel plot: Somnolence as treatment emergent adverse effect** 52](#_Toc191285999)

[**10.2.** **Trim-and- fill funnel plots: Primary outcomes with at least 10 studies** 53](#_Toc191286000)

[**10.2.1.** **Trim-and-fill plot: Somnolence as treatment emergent adverse effect** 53](#_Toc191286001)

[**10.3.** **Egger’s regression test for funnel plot asymmetry: Primary outcomes with at least 10 studies** 54](#_Toc191286002)

[**10.3.1.** **Egger’s regression test for funnel plot asymmetry: Somnolence as treatment emergent adverse effect** 54](#_Toc191286003)

[11. **Head-to-head comparisons of agomelatine with other antidepressants** 55](#_Toc191286004)

[12. **References** 56](#_Toc191286005)


# **PRISMA Checklist**

**Table S1** PRISMA Checklist

| Section /Topic | Item # | Checklist item | Reported on Page # |
| --- | --- | --- | --- |
| TITLE | | | |
| Title | 1 | Identify the report as a systematic review, meta-analysis, or both. . | 1-2 |
| ABSTRACT | | | |
| Abstract | 2 | Provide a structured summary including, as applicable: background; objectives; data sources; study eligibility criteria, participants, and interventions; study appraisal and synthesis methods; results; limitations; conclusions and implications of key findings; systematic review registration number. | 3 |
| INTRODUCTION | | | |
| Rationale | 3 | Describe the rationale for the review in the context of what is already known. | 4-6 |
| Objectives | 4 | Provide an explicit statement of questions being addressed with reference to participants, interventions, comparisons, outcomes, and study design (PICOS). | 6 |
| METHODS | | | |
| Protocol and registration | 5 | Indicate if a review protocol exists, if and where it can be accessed (e.g., Web address), and, if available, provide registration information including registration number. | 6 & supp 2; 3 |
| Eligibility criteria | 6 | Specify study characteristics (e.g., PICOS, length of follow-up) and report characteristics (e.g., years considered, language, publication status) used as criteria for eligibility, giving rationale. | 7-8 |
| Information sources | 7 | Describe all information sources (e.g., databases with dates of coverage, contact with study authors to identify additional studies) in the search and date last searched. | 9 & supp 4 |
| Search selection | 8 | Present full electronic search strategy for at least one database, including any limits used, such that it could be repeated. | 9 & supp 4 |
| Study selection | 9 | Specify the methods used to decide whether a study met the inclusion criteria of the review, including how many reviewers screened each record and each report retrieved, whether they worked independently, and if applicable, details of automation tools used in the process. | 7 |
| Data collection process | 10 | Describe method of data extraction from reports (e.g., piloted forms, independently, in duplicate) and any processes for obtaining and confirming data from investigators. | 9 |
| Data items | 11a | List and define all variables for which data were sought (e.g., PICOS, funding sources) and any assumptions and simplifications made. | 7-8 |
| Risk of bias in individual studies | 12 | Describe methods used for assessing risk of bias of individual studies (including specification of whether this was done at the study or outcome level), and how this information is to be used in any data synthesis. | 11 & supp 7 |
| Summary measures | 13 | State the principal summary measures (e.g., risk ratio, difference in means). | 9 |
| Synthesis of results | 14 | Describe the methods of handling data and combining results of studies, if done, including measures of consistency (e.g., I^2^) for each meta-analysis. | 10 |
| Risk of bias across studies | 15 | Specify any assessment of risk of bias that may affect the cumulative evidence (e.g., publication bias, selective reporting within studies). | 11 |
| Additional analyses | 16 | Describe methods of additional analyses (e.g., sensitivity or subgroup analyses, meta-regression), if done, indicating which were pre-specified. | 10-11 |
| RESULTS | | | |
| Study selection | 17 | Give numbers of studies screened, assessed for eligibility, and included in the review, with reasons for exclusions at each stage, ideally with a flow diagram. | 12-13; supp 7 |
| Study characteristics | 18 | For each study, present characteristics for which data were extracted (e.g., study size, PICOS, follow-up period) and provide the citations. | 12-13 and supp 8; 9 |
| Risk of bias in studies | 19 | Present data on risk of bias of each study and, if available, any outcome level assessment (see item 12). | 13 and supp 9 |
| Results of individual studies | 20 | For all outcomes considered (benefits or harms), present, for each study: (a) simple summary data for each intervention group (b) effect estimates and confidence intervals, ideally with a forest plot. | 13-18 and supp 11 |
| Synthesis of results | 21 | Present results of each meta-analysis done, including confidence intervals and measures of consistency. | 13-18 and supp 11 |
| Risk of bias across studies | 22 | Present results of any assessment of risk of bias across studies (see Item 15). | 19 and supp 12 |
| Additional analysis | 23 | Give results of additional analyses, if done (e.g., sensitivity or subgroup analyses, meta-regression [see Item 16]). | 19 and supp 11;13 |
| DISCUSSION | | | |
| Discussion | 24 | Summarize the main findings including the strength of evidence for each main outcome; consider their relevance to key groups (e.g., healthcare providers, users, and policy makers). | 19-22 |
| Limitations | 25 | Discuss limitations at study and outcome level (e.g., risk of bias), and at review-level (e.g., incomplete retrieval of identified research, reporting bias). | 22 |
| Conclusions | 26 | Provide a general interpretation of the results in the context of other evidence, and implications for future research. | 23 |
| FUNDING | | | |
| Funding | 24a | Describe sources of funding for the systematic review and other support (e.g., supply of data); role of funders for the systematic review. | 23 |

# **Protocol of the systematic review and meta-analysis**

## **Effects of Agomelatine on sleep: a systematic review and meta-analysis**

Ioannis Anastasiou, Andreas S. Lappas, Nikolaos Christodoulou, Myrto T. Samara, Panagiota Fallon

**Citation**

Ioannis Anastasiou, Andreas S. Lappas, Nikolaos Christodoulou, Myrto T. Samara, Panagiota Fallon. Effects of Agomelatine on sleep: a systematic review and meta-analysis. PROSPERO 2022 CRD42022385063 Available from: <https://www.crd.york.ac.uk/prospero/display_record.php?ID=CRD42022385063>

### **Review Question**

The objective of this systematic review and meta-analysis is to examine the efficacy and tolerability of Agomelatine in comparison with placebo on sleep-related outcomes, for patients with any health diagnosis.

**Searches**

The following sources will be searched without any restrictions in terms of language or publication period and status:

1. Electronic databases: multiple systematic searches will be conducted using MEDLINE (via Ovid), Embase, APA PsycINFO, the Cochrane Central Register of Controlled Trials (CENTRAL), the WHO International Clinical Trials Registry Platform (ICTRP) and the clinical trials register ClinicalTrials.gov.
2. Previous reviews: we will search previously published reviews for relevant studies.
3. Reference searching: we will manually review the references of all identified studies.

### **Types of Study to be Included**

We will only include randomized controlled trials (RCTs) examining the use of Agomelatine for any mental health and/or physical health diagnosis and reporting on any sleep-related efficacy and/or safety and tolerability outcomes. If the details of randomization are unclear (as per the Cochrane Handbook), we will include these trials, but then exclude them in a sensitivity analysis. We will include all relevant RCTs that meet our broad inclusion criteria irrespective of the use of blinding (open label, single-, or double-blind) and the reporting of usable data or not. There will be no language restriction to mitigate the risk of language bias and maximize the amount of usable data. We will exclude quasi-randomised studies, such as those that allocate intervention by alternate days of the week. The minimum duration of pharmacotherapy will be set at 5 days.

### **Condition or Domain Being Studied**

This review will be diagnosis-blind in terms of conditions being studied. Any health condition for which Agomelatine has been used as a treatment intervention in an eligible study will be examined.

### **Participants/Population**

Similarly, any population with health problems, irrespective of diagnosis will be included. There will be no restrictions in terms of age, gender and comorbidities.

### **Intervention(s), Exposure(s)**

The study will include any randomized placebo-controlled clinical trial that has used Agomelatine as an intervention, and has reported on any sleep-related efficacy and/or safety and tolerability.

### **Comparator(s)/Control**

Placebo.

### **Main Outcome(s)**

The main outcomes will be:

1. Total nocturnal sleep time
2. Sleep quality as measured by any validated sleep quality measure/questionnaire
3. Insomnia as a treatment emergent side effect
4. Somnolence as a treatment emergent side effect.

### **Measures of Effect**

We aim to divide all outcomes into immediate (up to 7 days), short term (up to 16 weeks) and long term (over 16 weeks). Immediate and short-term will be our primary outcomes. For dichotomous outcomes we will calculate a standard estimation of the risk ratio (RR) and its 95% confidence interval (CI). For continuous outcomes we will estimate the mean difference (MD) between groups. We prefer not to calculate effect size measures such as standardized mean difference (SMD). However if scales of significant similarity are used, we will presume there is a small difference in measurement, and we will calculate SMDs.

### **Additional Outcome(s)**

1. Sleep onset latency: the time required to fall asleep, which is a measure of sleep onset insomnia.
2. Number of nocturnal awakenings, which is a measure of disturbances in sleep continuity.
3. Nocturnal time spent awake following sleep onset, which is a quantitative measure of sleep maintenance.
4. Daytime impairment as measured by performance tasks and self-reported scales, for example the Epworth Sleepiness Scale or the Stanford Sleepiness Scale. This outcome is clinically very relevant as there is a clear link between sleep disturbances and daytime impairment due to this, leading to impairment of quality of life.
5. Patients’ subjective well-being/Quality of life (e.g. SF-36, EURO-Quol). This is another clinically relevant outcome combining elements of both efficacy and tolerability.
6. Polysomnographic or actigraphic recordings of the primary outcome “total nocturnal sleep time”. This will allow us to control for the potential differences between the patient-rated subjective and clinician-rated objective evaluation of insomnia.
7. Therapeutic effect on parasomnias (nightmares, vivid dreams, parasomnia behaviours, sleep disturbances where this is mentioned in general etc.).
8. Number of dropouts due to adverse effects.
9. Number of dropouts due to sleep-related adverse effects.
10. Total number of participants with adverse effects as a global measure of tolerability outcomes.
11. Total number of participants with sleep-related adverse effects as a global measure of sleep-related tolerability outcomes.
12. Number of participants that used an hypnotic rescue treatment for insomnia with a hypnotic drug during the trial.
13. Parasomnias (nightmares, vivid dreams, parasomnia behaviours, sleep disturbances where this is mentioned in general etc.) as treatment emergent side effects.
14. Any other sleep related outcome.

### **Measures of Effect**

We aim to divide all outcomes into immediate (up to 7 days), short term (up to 16 weeks) and long term (over 16 weeks). Immediate and short-term will be our primary outcomes. For dichotomous outcomes we will calculate a standard estimation of the risk ratio (RR) and its 95% confidence interval (CI). For continuous outcomes we will estimate the mean difference (MD) between groups. We prefer not to calculate effect size measures such as standardized mean difference (SMD). However if scales of significant similarity are used, we will presume there is a small difference in measurement, and we will calculate SMDs.

### **Data Extraction (Selection and Coding)**

At least two review authors will independently extract data from all included studies. Any conflicts will be discussed with a third author. If the disagreement is not resolved, we will attempt to contact the authors of the study for clarification. Change scores will be preferred over endpoint values.

### **Risk of Bias (Quality) Assessment**

At least two review authors will work independently to assess the risk of bias by using the Cochrane Risk of Bias Tool for Randomized Trials.

### **Strategy for Data Synthesis**

We will employ a random-effects model for the analyses (Der-Simonian 1986). We understand that there is no closed argument for preference of fixed or random effects model. The random-effects model is usually more conservative in terms of statistical significance, although as a disadvantage it puts added weight onto smaller studies which can either inflate or deflate the effect size. Therefore, we will examine in a sensitivity analysis whether using a fixed-model markedly changes the results of the primary outcomes.

All analyses will be on an intention-to-treat (ITT) basis. We will only use completed analyses if ITT data are unavailable. We will address this issue in the risk of bias in included studies tool. We will also conduct a sensitivity analysis by excluding studies which presented only completer data.

The effect size for dichotomous outcomes will be assessed by calculating the Risk Ratio (RR). The effect size for continuous outcomes will be assessed by calculating the weighted mean difference (MD). If data are presented indifferent scales, then the standardized mean difference (SMD) will be calculated instead. Missing standard deviations will be calculated from standard errors or estimated from confidence intervals, t-values, or p-values as described in section 7.7.3 of the Cochrane Handbook for Systematic Reviews.

Heterogeneity: All included trials will be considered initially. Heterogeneity will be investigated by inspection of the forest plots. Statistical heterogeneity will be tested with the I² test and quantified by the I² statistics. Potential reasons for heterogeneity will be explored with subgroup analyses, but these will only be conducted on the primary outcomes.

### **Analysis of Subgroups or Subsets**

Subgroup analysis will be conducted for the primary outcomes only (depending on the availability):

1. Per primary diagnosis
2. Participants with sleep disturbance symptoms vs. not. If possible, symptoms will be differentiated e.g. insomnia, nightmares, parasomnia etc.
3. Monotherapy vs. add-on drug treatment
4. Participants older than 65 vs. not.
5. Comorbid substance misuse vs. not.
6. Presence of an organic mental disorder vs. not.
7. Presence of a primary medical disorder vs. not.

### **Sensitivity Analyses:**

1. Exclusion of non-double- blind studies (open and single-blind studies)
2. Exclusion of studies that presented only completer analyses
3. Exclusion of studies with high risk of bias
4. Fixed effect instead of random effects model
5. Exclusion of studies with imputed data
6. Exclusion of sponsored studies
7. Exclusion of studies that allowed the use of hypnotics.

### **Contact Details for Further Information**

Ioannis Anastasiou

[johnanastasiou1999@gmail.com](mailto:johnanastasiou1999@gmail.com)

### **Organisational Affiliation of The Review**

Department of Psychiatry, School of Medicine, University of Thessaly

### **Review Team Members and Their Organisational Affiliations [1 change]**

Mr Ioannis Anastasiou. University of Thessaly

Dr Andreas S. Lappas. 1. Department of Geriatric Liaison Psychiatry, Royal Gwent Hospital, Newport, UK 2. Department of Psychiatry, School of Medicine, University of Thessaly, Greece

Assistant/Associate Professor Nikolaos Christodoulou. 1. Department of Psychiatry, School of Medicine, University of Thessaly, Greece 2. University of Nottingham, Medical School, UK

Assistant/Associate Professor Myrto T. Samara. Department of Psychiatry, School of Medicine, University of Thessaly

Ms Panagiota Fallon. University of Thessaly, Faculty of Medicine

### **Type and Method of Review**

Intervention, Meta-analysis, and Systematic review.

# **Deviations from the protocol.**

Although we preliminary decided utilising change values, we ultimately decided to analyse endpoint values instead. This approach allowed us to address any issues of missing values of SD for change scores.

# **Search strategy**

The search strategy for Ovid Medline is detailed below. Variations of this search strategy were tailored to meet the specific requirements of the additional databases, including (1) APA PsycInfo, (2) Cochrane Central Register of Controlled Trials (CENTRAL), and (3) Embase. APA PsychInfo, Embase and Medline were searched via Ovid. Additionally, the WHO International Clinical Trials Registry Platform (ICTRP) and the clinical trials register ClinicalTrials.gov were searched individually, using Agomelatine and all its variations as a keyword.

**Table S2** Ovid MEDLINE Search String

| Database: Ovid MEDLINE(R) ALL <1946 to February 16, 2025>  Search Strategy:  --------------------------------------------------------------------------------  1 randomized controlled trial.pt. (582235)  2 controlled clinical trial.pt. (95125)  3 randomized.ab. (584892)  4 placebo.ab. (233827)  5 drug therapy.fs. (2553906)  6 randomly.ab. (397137)  7 trial.ab. (626605)  8 groups.ab. (2444627)  9 1 or 2 or 3 or 4 or 5 or 6 or 7 or 8 (5529524)  10 exp animals/ not humans.sh. (5071725)  11 9 not 10 (4820254)  12 agomelatine.mp. (948)  13 ("N-(2-(7-methoxy-1-naphthyl)ethyl)acetamide" or "n (2 (7 methoxy 1 naphthyl)ethyl)acetamide" or "AGO 178" or "AGO-178" or "AGO178" or "Thymanax" or "Valdoxan" or "S20098" or "S 20098" or "S-20098" or "137R1N49AD").tw,kf. (109)  14 12 or 13 (957)  15 11 and 14 (508)  *************************** |
| --- |

# **PRISMA Flow Diagram**

**Figure S1** PRISMA 2020 Flow Diagram

**Identification of studies via databases and registers**

Records removed *before screening*:

- Duplicate records removed (n = 221)
- Records markers as ineligible by automation tools (n = 0)
- Records removed for other reasons (n = 0)

Records identified through databases and registries searching up to March 2025 (n = 1521)

**Identification**

Records excluded

(n = 1244

Records screened (n = 1300)

Reports sought for retrieval

(n = 56)

Reports not retrieved

(n = 0)

**Screening**

Reports excluded with reasons (n = 31):

- Comparison not relevant

(n = 17)

- Outcomes not relevant

(n = 6)

- Intervention not relevant (n = 5)
- Study design not relevant

(n = 3)

Reports assessed for eligibility

(n = 56)

Studies included in review

(n = 25)

**Included**

# **Table of Included Studies**

**Table S3** Included studies

| **Study ID** | **Study duration and design** | **Population characteristics** | **Intervention(s) and Comparator(s)** | **Key outcomes** |
| --- | --- | --- | --- | --- |
| Arango (2022) | 12-week, double-blind parallel RCT  **Location:**  Europe  Russia  South Africa  **Setting:**  Inpatient | **n=** 400  **Age (mean ± SD):**  13.7 ± 2.7 years  **Sex:**  62% females  **Diagnosis:**  MDD | **Intervention:**  Agomelatine, oral, dose 10 to 25mg/day, n= 197  **Comparator(s):**  Placebo, n= 103  Or  Fluoxetine, dose range 10 to 20mg/day, n= 100 (data not relevant)  **Add-on** (standardised manualised psychosocial counselling) | **Efficacy:**   - N.I.   **Safety:**   - Drop-outs due any AE - Dropouts due to any sleep-related AE - Any AE - Somnolence AE |
| Azadi 2024 | 12-week, double-blind parallel RCT  **Location:**  Iran  **Setting:**  Outpatients | **n=** 70  **Age (mean ± SD):**  34.30 ± 11.24 years  **Sex:**  55.7% females  **Diagnosis:**  MDD | **Intervention:**  Agomelatine, oral, dose 10 to 20mg/day, n= 35  **Comparator(s):**  Placebo, n= 35  **Add-on** (Escitalopram 10 to 20 mg/day) | **Efficacy:**   - N.I.   **Safety:**   - Dropouts due to any sleep-related AE - Any AE - Insomnia AE |
| Ballester (2019) | 24-week, triple-blind crossover RCT  **Location**:  Spain  **Setting:**  Inpatient | **n=** 50  **Age (mean ± SD):**  35.00 ± 12.00 years  **Sex:**  83% males  **Diagnosis:**  ASD | **Intervention:**  Agomelatine, oral, dose 25mg/day, n= 25  **Comparator:**  Placebo, n= 25  **Monotherapy** | **Efficacy:**   - Total sleep time (ACM) - Sleep onset latency (ACM) - Sleep continuity (ACM) - Nocturnal time spent post awake (ACM)   **Safety:**   - Drop-outs due any AE - Any AE |
| CAGO 2302; NCT01110902; | 8-week, double-blind parallel RCT  **Location:**  USA  **Setting:**  Outpatient | **n=** 590  **Age (mean ± SD):**  42.10 ± 12.70 years  **Sex:**  66.30% females  **Diagnosis:**  MDD | **Intervention:**  Agomelatine, sublingual, dose 0.5 to 1mg/day, n= 387  **Comparator(s):**  Placebo, n= 203  **Monotherapy** | **Efficacy:**   - Sleep quality (LSEQ) - Sleep continuity (LSEQ) - Sleep onset latency (LSEQ) - Daytime impairment (LSEQ)   **Safety:**   - Drop-outs due any AE - Any AE - Any sleep-related AE - Insomnia AE - Somnolence AE |
| CL2-90098-009 | 8-week, double-blind parallel RCT  **Location**:  Canada  Europe  Mexico  Russia  **Setting:**  Outpatient | **n=** 557  **Age (mean ± SD):**  45.30 ± 13.00 years  **Sex:**  63.90% females  **Diagnosis:**  MDD | **Intervention:**  Agomelatine, sublingual, dose 0.25 to 1mg/day, n= 414  **Comparator(s):**  Placebo, n= 143  **Monotherapy** | **Efficacy:**   - N.I.   **Safety:**   - Drop-outs due to any AE - Any AE |
| Heun (2013) | 8-week, double-blind parallel RCT  **Location:**  Europe  South America  **Setting:**  Outpatient | **n=** 222  **Age (mean ± SD):**  71.80 ± 5.00 years  **Sex:**  68% females  **Diagnosis:**  MDD | **Intervention:**  Agomelatine, oral, dose 25 to 50mg/day, n=151  **Comparator(s):**  Placebo, n= 71  **Monotherapy** | **Efficacy:**   - N.I.   **Safety:**   - Drop-outs due to any AE - Any AE - Any sleep-related AE - Somnolence AE |
| Kennedy (2006) | 6-week, double-blind parallel RCT  **Location**:  Canada  Europe  South Africa  **Setting:**  Outpatient | **n=** 212  **Age (mean ± SD):**  42.50 ± 12.50 years  **Sex:**  60.20 % females  **Diagnosis:**  MDD | **Intervention:**  Agomelatine, oral, 20 to 50mg/day, n= 106  **Comparator(s):**  Placebo, n= 105  **Monotherapy** | **Efficacy:**   - N.I.   **Safety:**   - Drop-outs due to any AE - Any AE - Any sleep-related AE - Insomnia AE |
| Kennedy (2014) | 6-week, double-blind parallel RCT  **Location**:  Europe  Russia  South America  **Setting:**  Outpatient | **n=** 549  **Age (mean ± SD):**  45.00 ± 12.56 years  **Sex:**  73.04% females  **Diagnosis:**  MDD | **Intervention:**  Agomelatine, oral, 10 to 50mg/day, n= 408  **Comparator(s):**  Placebo, n= 141  **Monotherapy** | **Efficacy:**   - N.I.   **Safety:**   - Drop-outs due to any AE - Any AE - Any sleep-related AE - Insomnia AE - Somnolence AE |
| Leproult (2005) | 4-week, double-blind cross-over RCT  **Location**:  Brussels  **Setting:**  Inpatient | **n=** 16  **Age (mean ± SD):**  60.00 ± N.I. years  (range 51 to 76 years)  **Sex:**  100% males  **Diagnosis:**  Healthy | **Intervention:**  Agomelatine, oral, dose 50mg/day, n= 8  **Comparator(s):**  Placebo, n= 8  **Monotherapy** | **Efficacy:**   - Total sleep time (PSG) - Sleep onset latency (PSG) - Sleep continuity (PSG) - Nocturnal time spent post awake (PSG)   **Safety:**   - N.I. |
| Loo (2002) | 8-week, double-blind parallel RCT  **Location**:  Europe  **Setting:**  N.I. | **n=** 711  **Age (mean ± SD):**  42.30 ± N.I. years  **Sex:**  66.50% males  **Diagnosis:**  MDD | **Intervention:**  Agomelatine, oral, dose 1 to 25mg/day, n= 425  **Comparator(s):**  Placebo, n= 139  Or  Paroxetine, dose 20mg/day, n= 147 (data not relevant)  **Monotherapy** | **Efficacy:**   - N.I.   **Safety:**   - Drop-outs due to any AE - Any AE - Any sleep-related AE - Insomnia AE - Somnolence AE |
| Mahdavi (2022) | 8-week, double-blind parallel RCT  **Location**:  Iran  **Setting:**  Outpatient | **n=** 46  **Age (mean ± SD):**  45.40 ± 12.90 years  **Sex:**  71% males  **Diagnosis:**  Chronic low back pain | **Intervention:**  Agomelatine, oral, dose 25mg/day, n= 23  **Comparator(s):**  Placebo, n= 23  **Add on** (Pregabalin 150mg/day) | **Efficacy:**   - N.I.   **Safety:**   - Drop-outs due any AE - Any AE - Any sleep-related AE |
| Montejo (2015) | 9-week, double-blind parallel RCT  **Location:**  **UK**  **Setting:**  N.I. | **n=** 133  **Age (mean ± SD):**  23.26 ± 4.35 years  **Sex:**  50.38% females  **Diagnosis:**  Healthy | **Intervention:**  Agomelatine, oral, dose 25 to 50mg/day, n= 65  **Comparator(s):**  Placebo, n= 32  Or  Escitalopram, dose 10mg/day, n= 36 (data not relevant)  **Monotherapy** | **Efficacy:**   - N.I.   **Safety:**   - Drop-outs due to any AE - Any AE - Any sleep-related AE - Somnolence AE |
| Nejati (2024) | 12-week, double-blind parallel RCT  **Country:** Iran  **Setting:** Outpatient | **n=** 60  **Age (mean ± SD):**  37.01 ± 3.21 years  **Sex:**  55.0% males  **Diagnosis:**  OCD | **Intervention:**  Agomelatine, oral, dose 50/day, n= 30  **Comparator(s):**  Placebo, n= 30  **Monotherapy** | **Efficacy:**   - N.I.   **Safety:**  Any AE |
| Olie (2007) | 6-week, double-blind parallel RCT  **Country**:  Europe  **Setting:**  Inpatient  Outpatient | **n=** 238  **Age (mean ± SD):**  45.00 ± 11.30 years  **Sex:**  73.50% females  **Diagnosis:**  MDD | **Intervention:**  Agomelatine, oral, dose 25 to 50mg/day, n= 118  **Comparator(s):**  Placebo, n= 120  **Monotherapy** | **Efficacy:**   - N.I.   **Safety:**   - Drop-outs due any AE - Any AE - Any sleep-related AE |
| Rouillon (2008) | 8-week, double-blind parallel RCT  **Country**:  Europe  **Setting:**  Outpatient | **n=** 267  **Age (mean ± SD):**  47.00 ± 12.90 years  **Sex:**  68.30% females  **Diagnosis:**  MDD | **Intervention:**  Agomelatine, sublingual, dose 1 to 2mg/day, n= 175  **Comparator(s):**  Placebo, n= 92  **Monotherapy** | **Efficacy:**   - Sleep quality (LSEQ) - Sleep continuity (LSEQ) - Sleep onset latency (LSEQ) - Daytime impairment (LSEQ)   **Safety:**   - Drop-outs due to any AE - Any AE - Any sleep-related AE - Somnolence AE |
| Salin (2019) | 12-week, double-blind parallel RCT  **Country**:  Thailand  **Setting:**  N.I. | **n=** 21  **Age (mean ± SD):**  N.I. ± N.I. years  **Sex:**  N.I.  **Diagnosis:**  Systemic lupus erythematosus | **Intervention:**  Agomelatine, oral, dose N.I mg/day, n= 10  **Comparator(s):**  Placebo, n= 11  **Monotherapy** | **Efficacy:**   - Sleep quality (PSQI)   **Safety:**   - Drop-outs due to any AE |
| Shokrani (2023) | 12-week, double-blind parallel RCT  **Country**:  Iran  **Setting:**  Outpatient | **n=** 65  **Age (mean ± SD):**  37.22. ± 12.81 years  **Sex:**  64% females  **Diagnosis:**  OCD | **Intervention:**  Agomelatine, oral, dose 25mg/day, n= 33  **Comparator(s):**  Placebo, n= 32  **Add-on** (Sertraline 100 to 800mg/day) | **Efficacy:**   - N.I.   **Safety:**   - Any AE - Any sleep-related AE - Insomnia AE - Somnolence AE |
| Stahl (2010) | 8-week, double-blind parallel RCT  **Country**: USA  **Setting:** N.I. | **n=** 503  **Age (mean ± SD):**  43.30 ± 12.28 years  **Sex:**  65.40% females  **Diagnosis:**  MDD | **Intervention:**  Agomelatine, oral, dose 25 to 50mg/day, n= 337  **Comparator(s):**  Placebo, n= 166  **Monotherapy** | **Efficacy:**   - Sleep quality (LSEQ) - Sleep onset latency (LSEQ)   **Safety:**   - Drop-outs due to any AE - Any AE - Any sleep-related AE - Somnolence AE |
| Stein (2008) | 12-week, double-blind parallel RCT  **Country**:  Europe  South Africa  **Setting:**  Outpatient | **n=** 121  **Age (mean ± SD):**  41.70 ± 12.20 years  **Sex:**  68.60% females  **Diagnosis:**  GAD | **Intervention:**  Agomelatine, oral, dose 25 to 50mg/day, n= 63  **Comparator(s):**  Placebo, n= 58  **Monotherapy** | **Efficacy:**   - Sleep quality (LSEQ) - Sleep continuity (LSEQ) - Sleep onset latency (LSEQ)   **Safety:**   - Drop-outs due to any AE - Any AE |
| Stein (2012) | 26-week, double-blind parallel RCT  **Country**:  Canada  Europe  **Setting:**  Outpatient. | **n=** 228  **Age (mean ± SD):**  46.40 ± 14.60 years  **Sex:**  62.30% females  **Diagnosis:**  GAD | **Intervention:**  Agomelatine, oral, dose 25 to 50mg/day, n= 114  **Comparator(s):**  Placebo, n= 114  **Monotherapy** | **Efficacy:**   - Sleep quality (LSEQ) - Sleep continuity (LSEQ) - Sleep onset latency (LSEQ) - Daytime impairment (LSEQ)   **Safety:**   - Drop-out due to any AE - Any AE |
| Stein (2014) | 12-week, double-blind parallel RCT  **Country**:  Europe  Russia  South America  South Korea  **Setting:**  Outpatient | **n=** 412  **Age (mean ± SD):**  42.60 ± 12.40 years  **Sex:**  71.60% females  **Diagnosis:**  GAD | **Intervention:**  Agomelatine, oral, dose 25 to 50mg/day, n= 139  **Comparator(s):**  Placebo, n= 131  Or  Escitalopram, dose 10mg/day, n= 139 (data not relevant)  **Monotherapy** | **Efficacy:**   - Sleep quality (LSEQ) - Sleep continuity (LSEQ) - Sleep onset latency (LSEQ) - Daytime impairment (LSEQ)   **Safety:**   - Drop-outs due to any AE - Any AE - Any sleep-related AE - Somnolence AE |
| Stein (2017) | 12-week, double-blind parallel RCT  **Country**:  Europe  Russia  **Setting:**  Outpatient | **n=** 412  **Age (mean ± SD):**  43.90 ± 13.90 years  **Sex:**  67.70% females  **Diagnosis:**  GAD | **Intervention:**  Agomelatine, oral, dose 10 to 25mg/day, n= 270  **Comparator(s):**  Placebo, n= 142  **Monotherapy** | **Efficacy:**   - N.I.   **Safety:**   - Drop-outs due to any AE - Any AE - Any sleep-related AE - Somnolence AE |
| Yatham (2016) | 8-week, double-blind parallel RCT  **Country**:  Australia  Canada  Europe  India  South Africa  South America  South Korea  **Setting:**  Outpatient | **n=** 344  **Age (mean ± SD):**  45.20 ± 12.60 years  **Sex:**  61.10% females  **Diagnosis:**  BD in current MDD episode | **Intervention:**  Agomelatine, oral, dose 25 to 50mg/day, n= 172  **Comparator(s):**  Placebo, n= 172  **Add-on** (lithium or valproate) | **Efficacy:**   - N.I.   **Safety:**   - Drop-outs due to any AE - Any AE - Any sleep related AE - Insomnia AE - Somnolence AE |
| Zajecka (2010) | 8-week, double-blind parallel RCT  **Country**:  USA  **Setting:**  Outpatient | **n=** 511  **Age (mean ± SD):**  43.80 ± 12.22 years  **Sex:**  66.70% females  **Diagnosis:**  MDD | **Intervention:**  Agomelatine, oral, dose 25 to 50mg/day, n= 338  **Comparator(s):**  Placebo, n= 173  **Monotherapy** | **Efficacy:**   - Sleep quality (LSEQ) - Sleep onset latency (LSEQ)   **Safety:**   - Drop-outs due to any AE - Any AE - Any sleep-related AE - Insomnia AE - Somnolence AE |
| Zohar (2010) | 16-week, double-blind parallel RCT  **Country**:  Europe  Israel  **Setting:**  Outpatient | **n=** 74  **Age (mean ± SD):**  39.00 ± 12.00 years  **Sex:**  51.4% males  **Diagnosis:**  OCD | **Intervention:**  Agomelatine, oral, dose 25 to 50mg/day, n= 39  **Comparator(s):**  Placebo, n= 35  **Monotherapy** | **Efficacy:**   - Sleep quality (LSEQ) - Sleep continuity - Sleep onset latency (LSEQ - Daytime impairment (LSEQ))   **Safety:**   - Drop-outs due to any AE - Any AE - Any sleep-related AE |
| **Abbreviations:**  **AE:** adverse effect; **ACM:** ambulatory circadian monitoring; **ASD:** Autism Spectrum Disorder; **BD:** Bipolar Disorder; **CAPS:** Clinician Administered Post-Traumatic Stress Disorder Scale; **GAD:** Generalised Anxiety Disorder; **LSEQ:** Leeds Sleep Evaluation Questionnaire; **MDD:** Major Depressive Disorder; **N.I.:** no information; **OCD:** obsessive-compulsive disorder; **PSG:** Polysomnography; **PSQI:** Pittsburgh Sleep Quality Index; **RCT:** randomised controlled trial; **SD:** standard deviation; **UK:** United Kingdom; **USA:** United States of America | | | | |

# **Risk of bias**

**Table S4.** Formula for assessment of overall Risk of Bias

| Global rating | Items with high risk | Items with unclear risk |
| --- | --- | --- |
| low risk | 0 | =< 3 |
| moderate | 0 | >3 |
| moderate | 1 | any |
| high risk | >1 | any |

**Figure S2.** Risk of bias summary plot


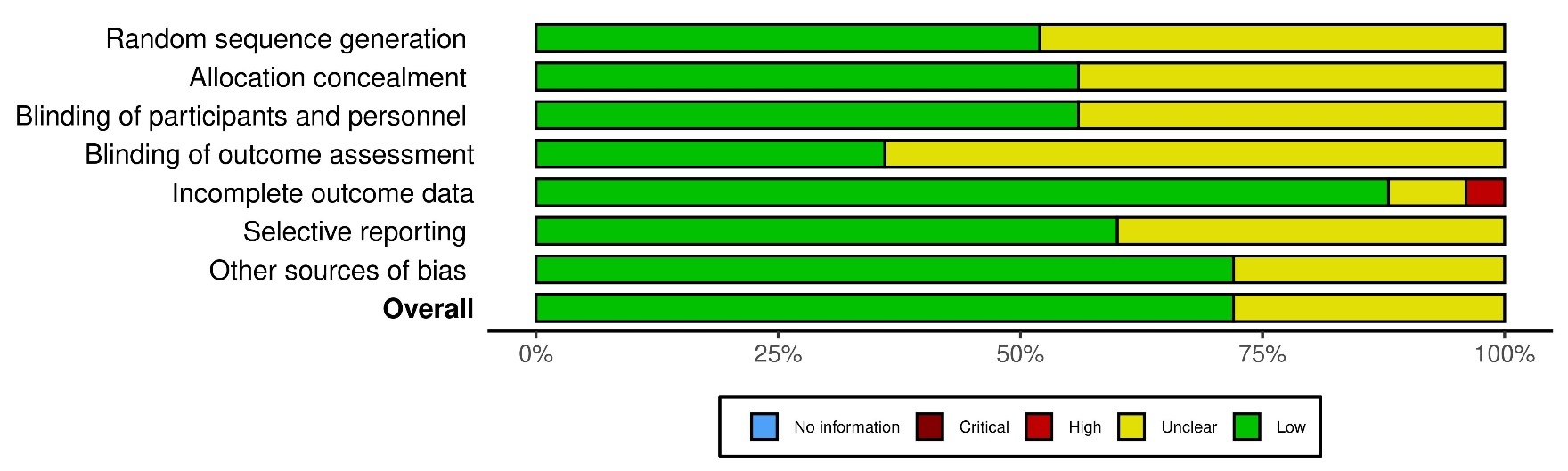


**Figure S 3.** Risk of bias assessment for individual studies


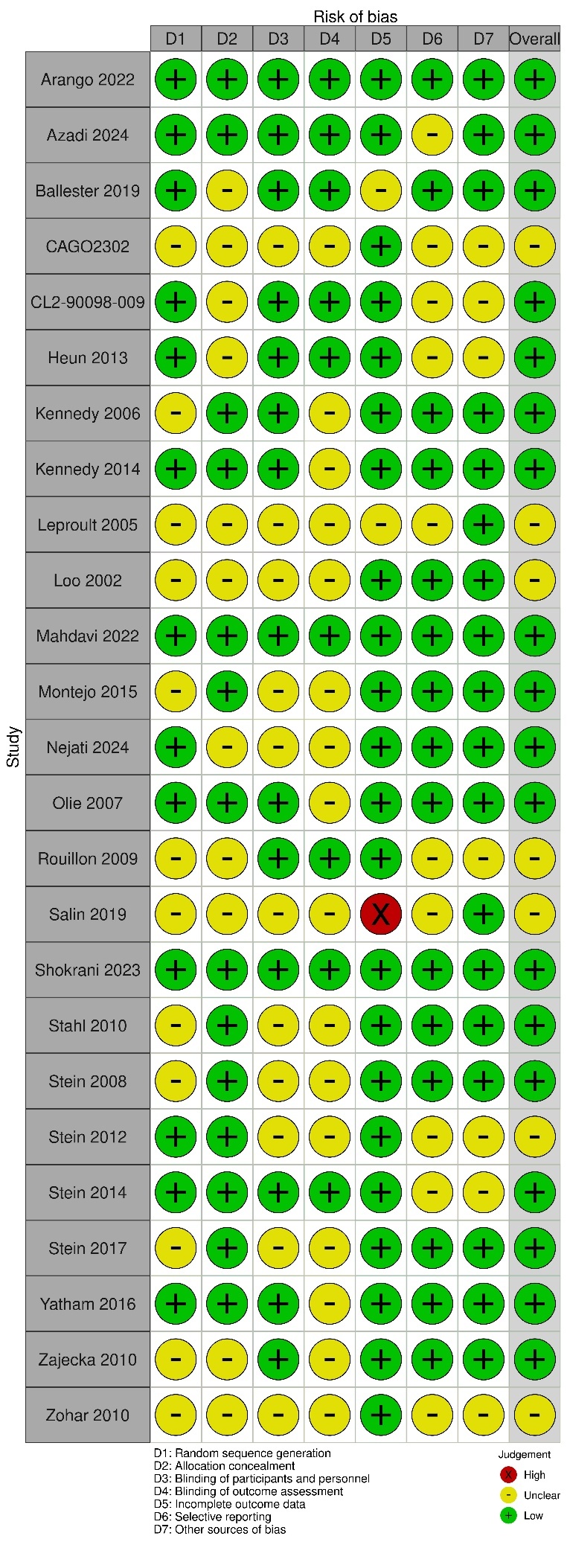


# **Ongoing Studies**

**Table S5** List of eligible ongoing studies

| **Study Registration/ID** | **Study Information & Status** | **Research questions & outcome measures** |
| --- | --- | --- |
| [ChiCTR1800018822](https://www.chictr.org.cn/showproj.aspx?proj=30321) | **Study design:**  RCT; multi-centre; parallel; double-blind  **Target recruitment:**  300 participants  **Country:**  China  **Status:**  Recruiting  **Estimated primary completion date:**  N.I. | **Population:**  Chronic insomnia and mild depression  **Intervention:**   - Agomelatine and CBTI - Zolpidem   **Comparator:**   - Placebo   **Relevant Outcomes:**   - Sleep quality (PSG) - Sleep awakening (PSG) - Daytime impairment (PSG) - Insomnia rebound during 6-12 mos |
| [ChiCTR2000032518](https://www.chictr.org.cn/showprojEN.html?proj=50671) | **Study design:**  RCT; single-centre; double-blind; parallel  **Target recruitment:**  120 participants  **Country:**  China  **Status:**  Recruiting  **Estimated primary completion date:**  N.I. | **Population:**  MDD  **Intervention:**   - Agomelatine and SRRIs   **Comparator:**   - Escitalopram - Sertraline   **Relevant Outcomes:**   - Potential sleep-related AE - Potential sleep-related drop-outs |

| **Study Registration/ID** | **Study Information & Status** | **Research questions & outcome measures** |
| --- | --- | --- |
| [ChiCTR2100046289](https://www.chictr.org.cn/showprojEN.html?proj=126182) | **Study design:**  RCT; single-centre; parallel; double-blind  **Target recruitment:**  50 participants  **Country:**  China  **Status:**  Recruiting  **Estimated primary completion date:**  N.I. | **Population:**  Epilepsy and MDD  **Intervention:**   - Agomelatine 25mg - Agomelatine 50mg   **Comparator:**   - Placebo   **Relevant Outcomes:**   - Sleep quality (sleep diary) |
| [ChiCTR2100048875](https://www.chictr.org.cn/showprojEN.html?proj=130377) | **Study design:**  RCT; multi-centre; parallel; double-blind  **Target recruitment:**  50 participants  **Country:**  China  **Status:**  Recruiting pending  **Estimated primary completion date:**  N.I. | **Population:**  RBD  **Intervention:**   - Agomelatine   **Comparator:**   - Placebo   **Relevant Outcomes:**   - Sleep quality (RBDQ-HK) - RWA (PSG) |

| **Study Registration/ID** | **Study Information & Status** | **Research questions & outcome measures** |
| --- | --- | --- |
| [CTRI/2011/08/001946](https://www.ctri.nic.in/Clinicaltrials/pmaindet2.php?EncHid=MjU2OQ==&Enc=&userName=agomelatine) | **Study design:**  RCT; multi-centre; parallel; open-label  **Target recruitment:**  200 participants  **Country:**  India  **Status:**  Open to recruitment  **Estimated primary completion date:**  N.I. | **Population:**  MDD  **Intervention:**   - Agomelatine   **Comparator:**   - Placebo   **Relevant Outcomes:**   - Sleep quality (LSEQ) - Sleep onset latency (LSEQ) |
| [EUCTR2013-003370-27-DE](https://www.clinicaltrialsregister.eu/ctr-search/search?query=eudract_number:2013-003370-27) | **Study design:**  RCT; single-centre; double-blind; parallel  **Target recruitment:**  80 participants  **Country:**  Germany  **Status:**  Completed (results not posted yet)  **Estimated primary completion date:**  N.I. | **Population:**  MDD  **Intervention:**   - Agomelatine - Escitalopram - Mirtazapine   **Comparator:**   - Placebo   **Relevant Outcomes:**   - Potential sleep-related AE - Potential sleep-related drop-outs |

| **Study Registration/ID** | **Study Information & Status** | **Research questions & outcome measures** |
| --- | --- | --- |
| [IRCT20170608034390N10](https://trialsearch.who.int/Trial2.aspx?TrialID=IRCT20170608034390N10) | **Study design:**  RCT; double-blind; parallel  **Target recruitment:**  52 participants  **Country:**  Iran  **Status:**  Recruiting pending  **Estimated primary completion date:**  N.I. | **Population:**  Alzheimer’s disease dementia  **Intervention:**   - Agomelatine (add-on)   **Comparator:**   - Placebo (add-on)   **Relevant Outcomes:**   - Potential sleep-related AE - Potential sleep-related drop-outs |
| [IRCT20230303057599N1](https://trialsearch.who.int/Trial2.aspx?TrialID=IRCT20230303057599N1); published protocol | **Study design:**  RCT; triple-blind; parallel  **Target recruitment:**  50 participants  **Country:**  Iran  **Status:**  Recruiting complete  **Estimated primary completion date:**  N.I. | **Population:**  Episodic migraine without aura  **Intervention:**   - Agomelatine   **Comparator:**   - Vitamin B1 (placebo)   **Relevant Outcomes:**   - Potential sleep-related AE - Potential sleep-related drop-outs |

| **Study Registration/ID** | **Study Information & Status** | **Research questions & outcome measures** |
| --- | --- | --- |
| [IRCT20220508054780N4](https://trialsearch.who.int/Trial2.aspx?TrialID=IRCT20220508054780N4) | **Study design:**  RCT; double-blind; parallel  **Target recruitment:**  74 participants  **Country:**  Iran  **Status:**  Recruiting pending  **Estimated primary completion date:**  N.I. | **Population:**  Staying more than 72 hours in the CICU  **Intervention:**   - Agomelatine   **Comparator:**   - Placebo   **Relevant Outcomes:**   - Potential sleep-related AE - Potential sleep-related drop-outs |
| [NCT04589143](https://clinicaltrials.gov/ct2/show/NCT04589143) | **Study design:**  RCT; multi-centre; double-blind; parallel  **Estimated Enrolment:**  137 participants  **Country:**  China  **Status:**  Completed (results not posted yet)  **Study Completion date:**  20-01-2023 | **Population:**  MDD  **Intervention:**   - Agomelatine   **Comparator:**   - Placebo   **Relevant Outcomes:**   - Severity of insomnia (AIS) |

| **Study Registration/ID** | **Study Information & Status** | **Research questions & outcome measures** |
| --- | --- | --- |
| [NCT05426304](https://clinicaltrials.gov/study/NCT05426304) | **Study design:**  RCT; multi-centre; double-blind; parallel  **Estimated Enrolment:**  420 participants  **Country:**  China  **Status:**  Not yet recruiting  **Estimated primary completion date:**  31-5-2024 | **Population:**  Poststroke depression  **Intervention:**   - Agomelatine   **Comparator:**   - Placebo   **Relevant Outcomes:**   - Sleep quality (PSQI) - Sleep continuity (ESS) |
| **Abbreviations:**  **AIS:** Athens Insomnia Scale; **CBTI:** Cognitive Behavioural Therapy for Insomnia; **CICU:** Cardiac surgery intensive care unit; **ESS**: Epworth Sleepiness Scale**; LSEQ:** Leeds Sleep Evaluation Questionnaire; **mos:** months; **MMD:** major depressive disorder; **N.I.:** no information; **OCD:** obsessive-compulsive disorder; **PSG**: Polysomnography; **PSQI:** Pittsburgh Sleep Quality Index; **REM:** rapid eye movement; **RBD**: REM sleep behaviour disorder; **RBDQ-HK:** REM Sleep Behavior Disorder Screening Questionnaire - Validation Study of the Hong Kong version; **RCT:** randomised controlled trial; **RWA:** REM without Atonia; **SRRIs:** selective serotonin reuptake inhibitors | | |

# **Forest plots for all outcomes**

## **Primary Outcomes**

### **Total Sleep Time (measured in minutes)**

**Figure S4.1.** Forest plot - Total sleep time measured in minutes, pooled result.


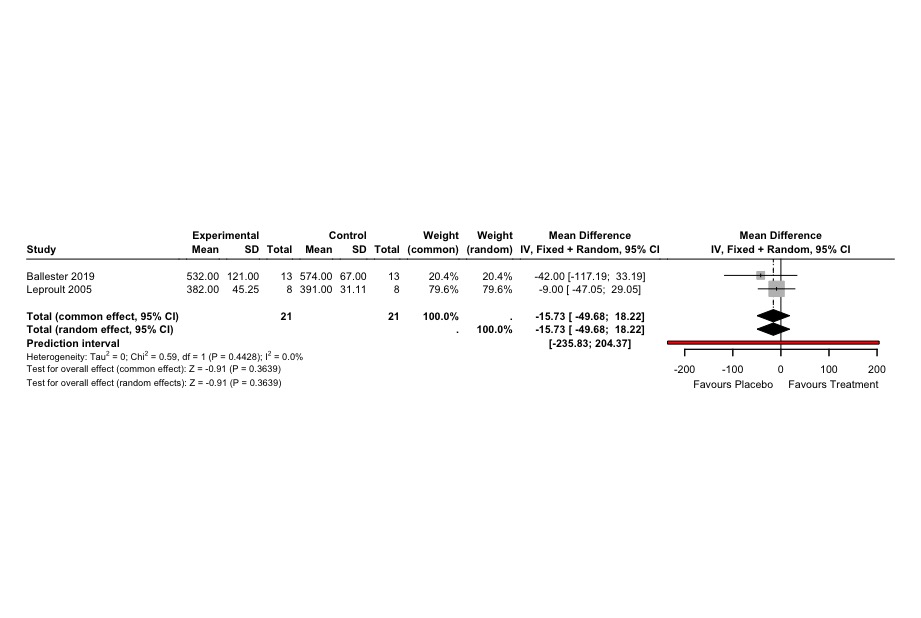


*Note.* MD = Weighted Mean Difference for TST with 95% CI (Confidence Intervals) and PI (Prediction Intervals).

**Figure S4.2.** Forest plot - Total sleep time measured in minutes, sub-group by diagnosis.


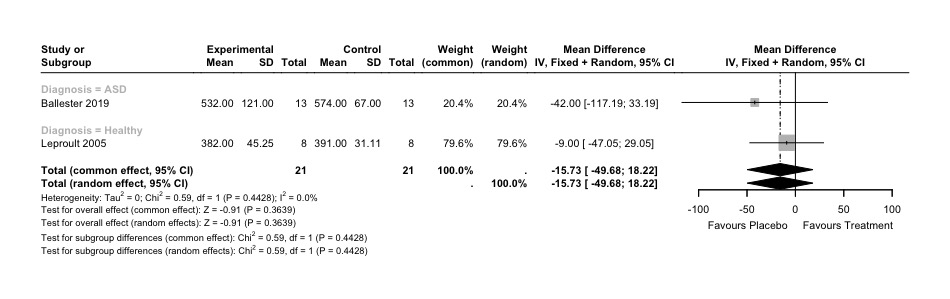


*Note.* MD = weighted mean difference for TST with 95% CI (Confidence Intervals).

**Figure S4.3.** Forest plot - Total sleep time measured in minutes, sub-group by presence of organic disorder.

**
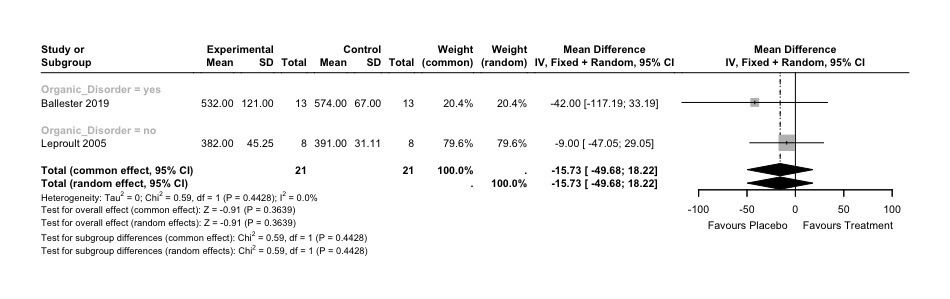
**

*Note.* MD = weighted mean difference for TST with 95% CI (Confidence Intervals).

**Figure S4.4.** Forest plot - Total sleep time measured in minutes, sensitivity analysis – excluding studies with use of hypnotics as rescue drug.

**
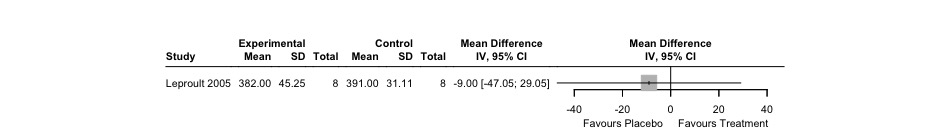
**

*Note.* MD = weighted mean difference for TST with 95% CI (Confidence Intervals).

### **Quality of Sleep (measured by PSQI and LSEQ)**

**Figure S5.1.** Forest plot – Quality of Sleep measured in PSQI or LSEQ, pooled result.


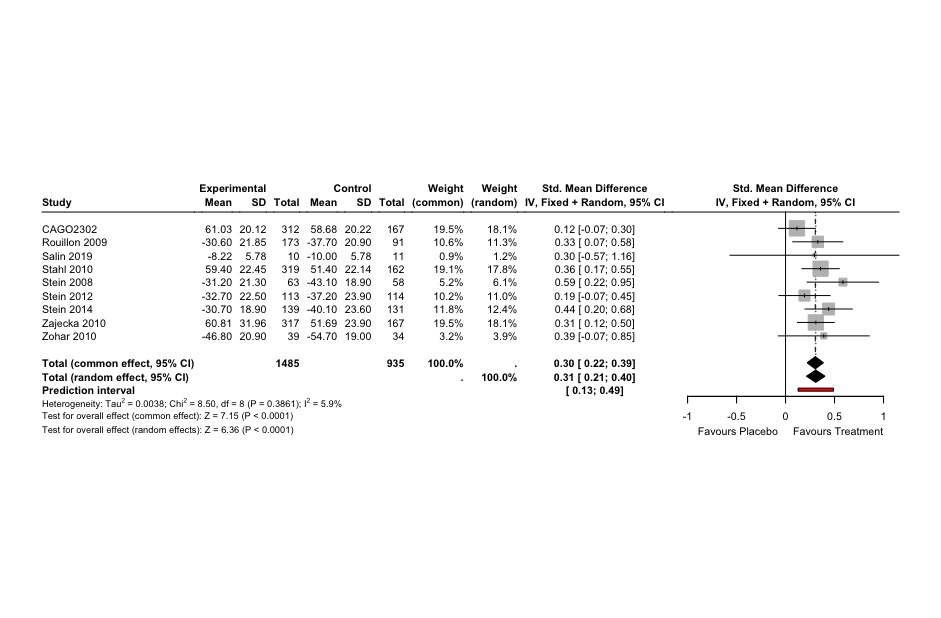


*Note.* SMD = standardized mean difference for Quality of Sleep (measured in LSEQ or PSQI) with 95% CI (Confidence Intervals) and PI (Prediction Intervals).

**Figure S5.2.** Forest plot – Quality of Sleep measured in PSQI or LSEQ, subgroup by diagnosis.

**
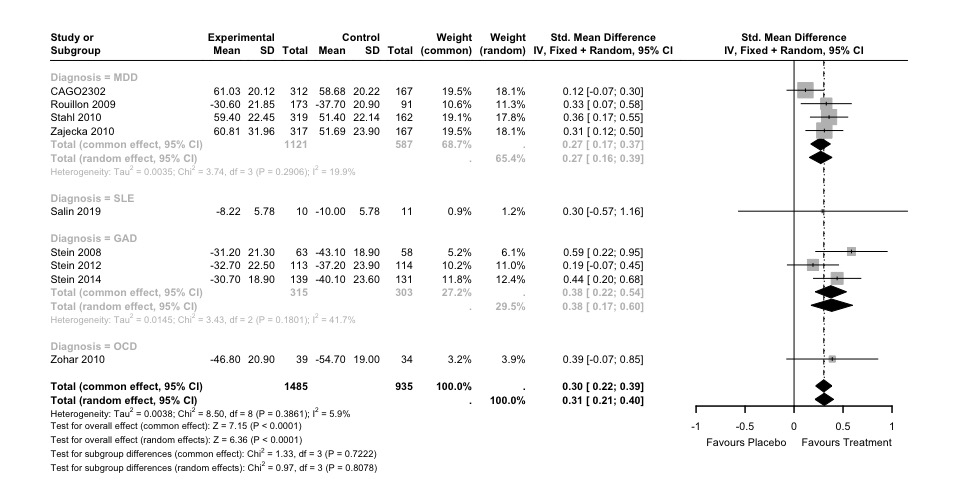
**

*Note.* SMD = standardized mean difference for Quality of Sleep (measured in LSEQ or PSQI) with 95% CI (Confidence Intervals).

**Figure S5.3.** Forest plot – Quality of Sleep measured in PSQI or LSEQ, subgroup by presence of organic disorder.

**
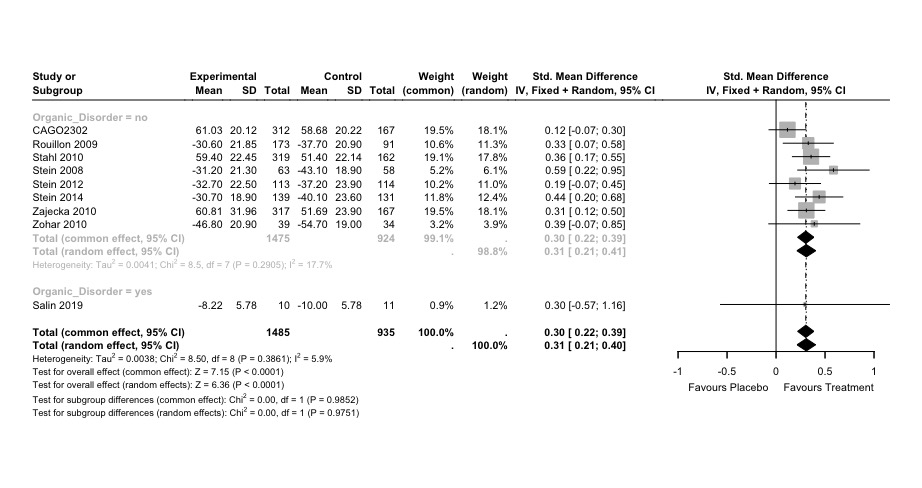
**

*Note.* SMD = standardized mean difference for Quality of Sleep (measured in LSEQ or PSQI) with 95% CI (Confidence Intervals).

**Figure S5.4.** Forest plot – Quality of Sleep measured in PSQI or LSEQ, subgroup by presence of primary medical disorder.


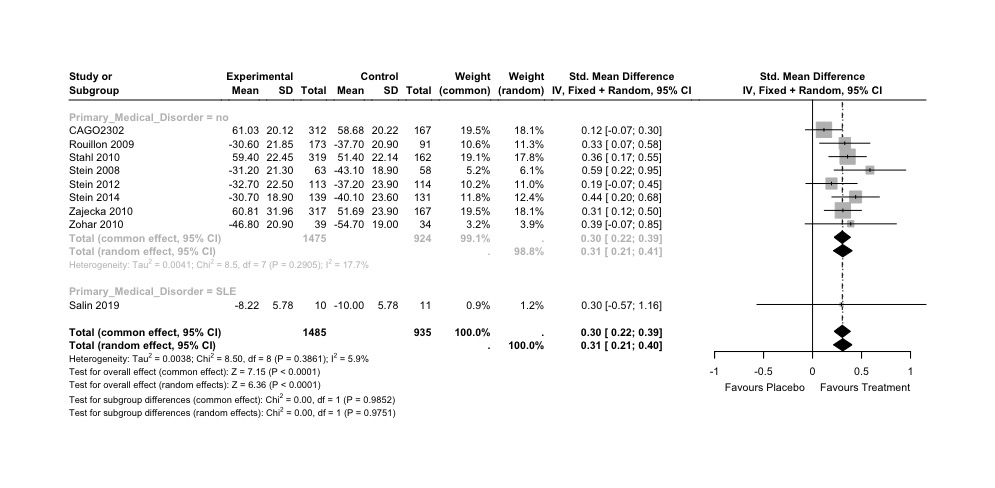


*Note.* SMD = standardized mean difference for Quality of Sleep (measured in LSEQ or PSQI) with 95% CI (Confidence Intervals).

**Figure S5.5.** Forest plot – Quality of Sleep measured in PSQI or LSEQ, Sensitivity analysis – excluding sponsored studies.


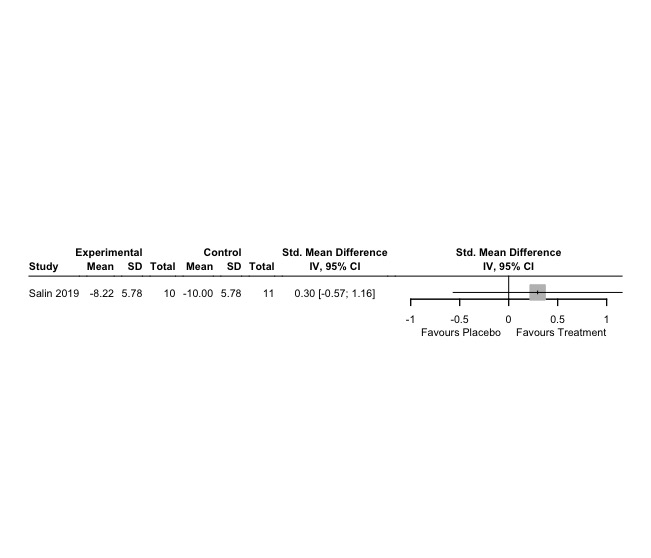


*Note.* SMD = standardized mean difference for Quality of Sleep (measured in LSEQ or PSQI) with 95% CI (Confidence Intervals).

### **Insomnia as treatment emergent adverse effect.**

**Figure S6.1.** Forest plot – Insomnia as a treatment emergent side effect, pooled result.


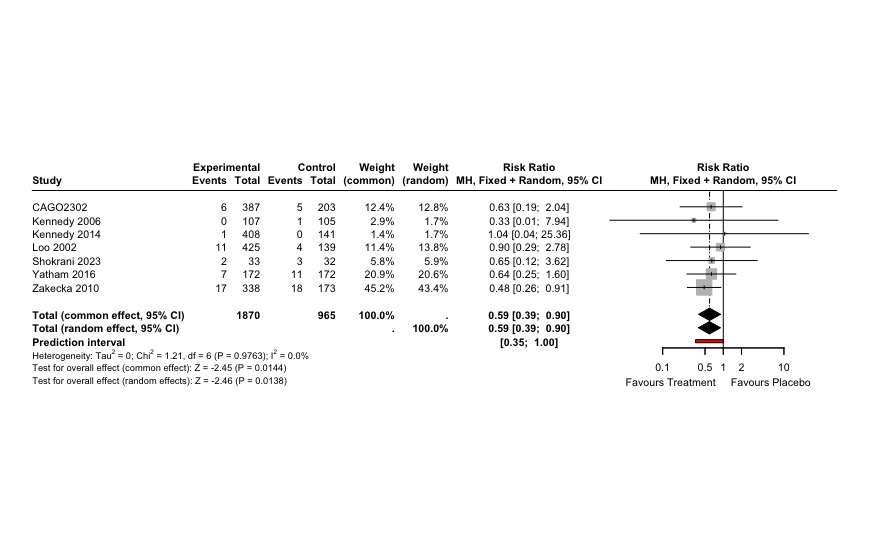


*Note.* RR = Risk Ratio for Insomnia as a treatment emergent side effect with 95% CI (Confidence Intervals) and PI (Prediction Intervals).

**Figure S6.2.** Forest plot – Insomnia as a treatment emergent side effect, subgroup by diagnosis.


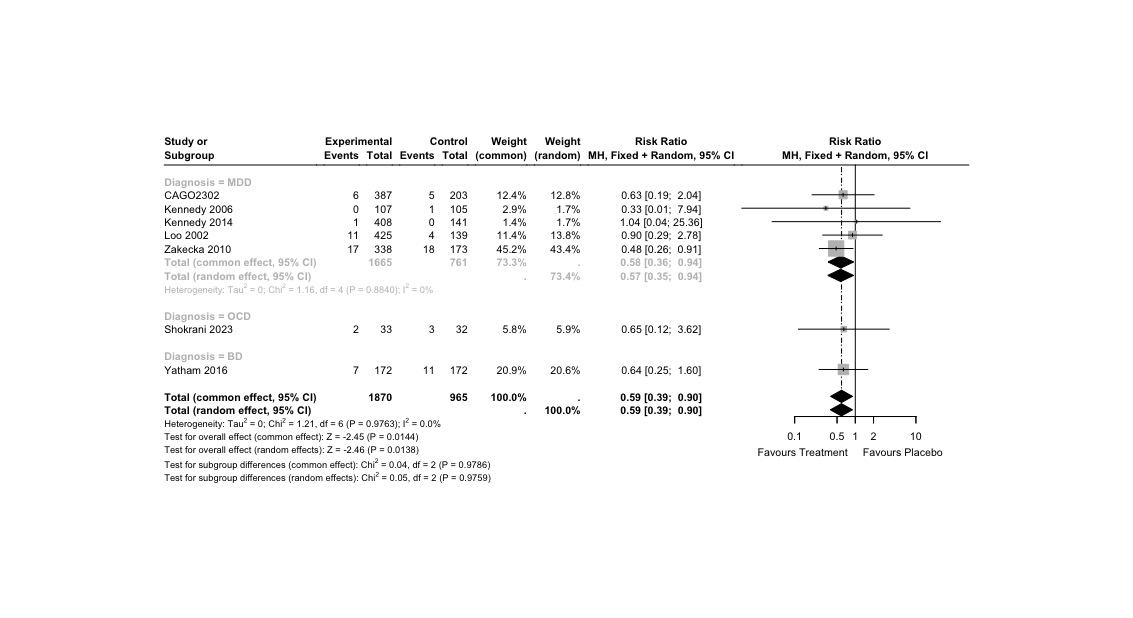


*Note.* RR = Risk Ratio for Insomnia as a treatment emergent side effect with 95% CI (Confidence Intervals).

**Figure S6.3.** Forest plot – Insomnia as a treatment emergent side effect, subgroup by use of add-on drug treatment.

**
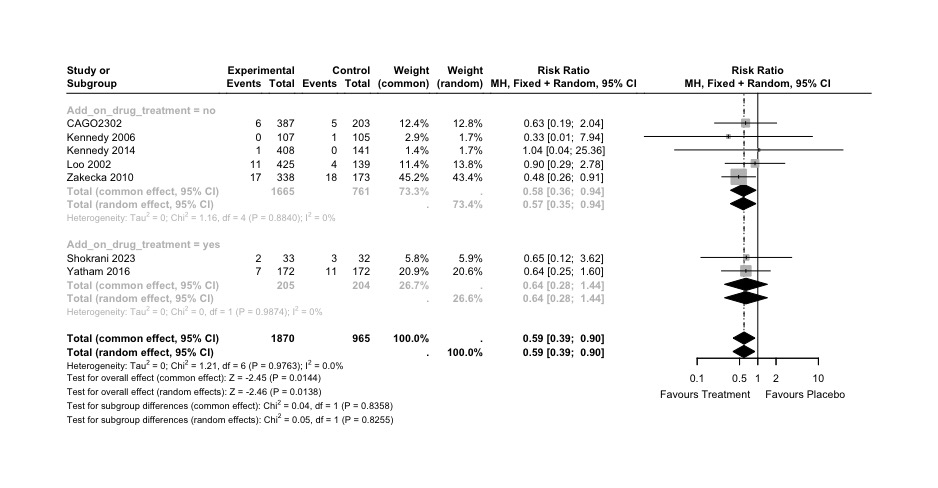
**

*Note.* RR = Risk Ratio for Insomnia as a treatment emergent side effect with 95% CI (Confidence Intervals).

**Figure S6.4.** Forest plot – Insomnia as a treatment emergent side effect, Sensitivity analysis – excluding sponsored studies.

**
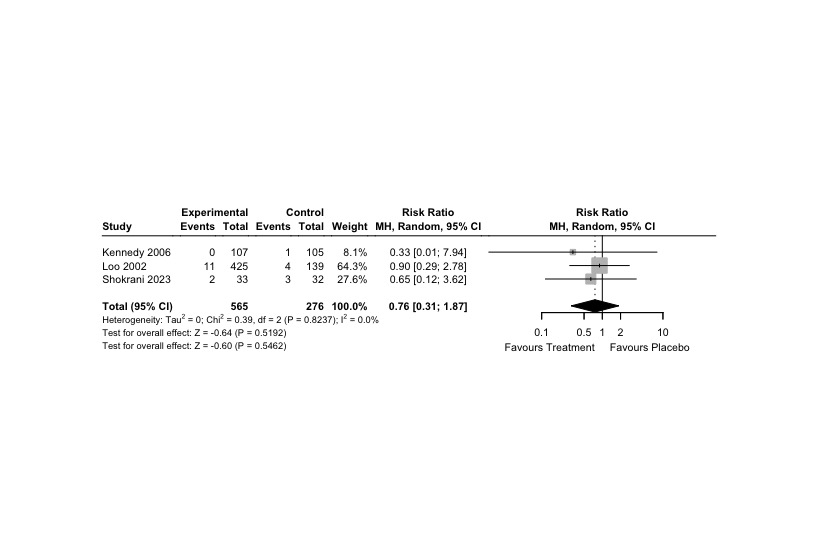
**

*Note.* RR = Risk Ratio for Insomnia as a treatment emergent side effect with 95% CI (Confidence Intervals).

**Figure S6.5.** Forest plot – Insomnia as a treatment emergent side effect, Sensitivity analysis – excluding studies using hypnotics.


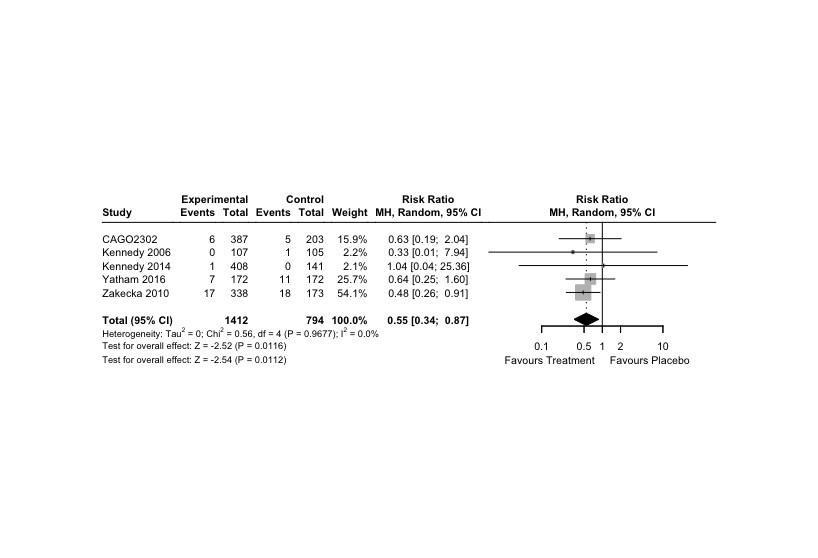


*Note.* RR = Risk Ratio for Insomnia as a treatment emergent side effect with 95% CI (Confidence Intervals).

### **Somnolence as treatment emergent adverse effect**

**Figure S7.1.** Forest plot - Somnolence as treatment emergent adverse effect, pooled result.

**
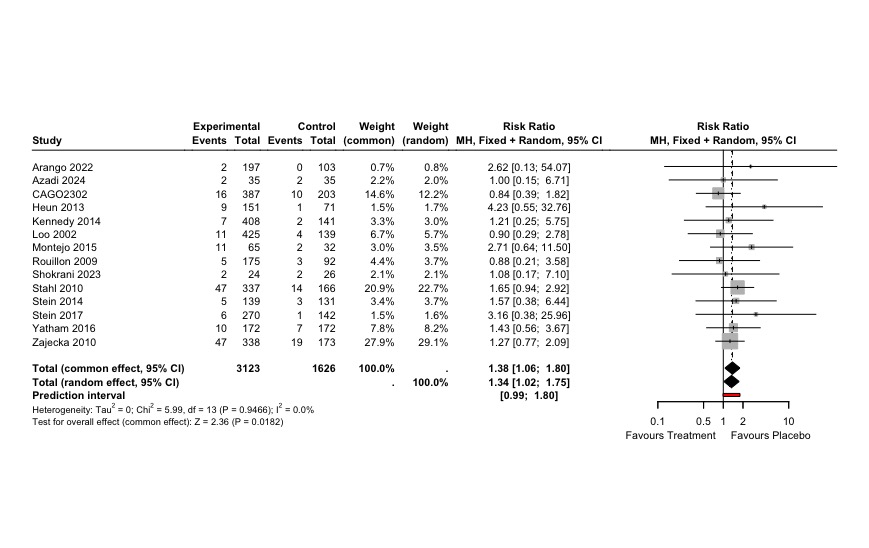
**

*Note.* RR = Risk Ratio for Somnolence as treatment emergent adverse effect with 95% CI (Confidence Intervals) and PI (Prediction Intervals).

**Figure 7.2.** Forest plot - Somnolence as treatment emergent adverse effect, subgroup by diagnosis.

**
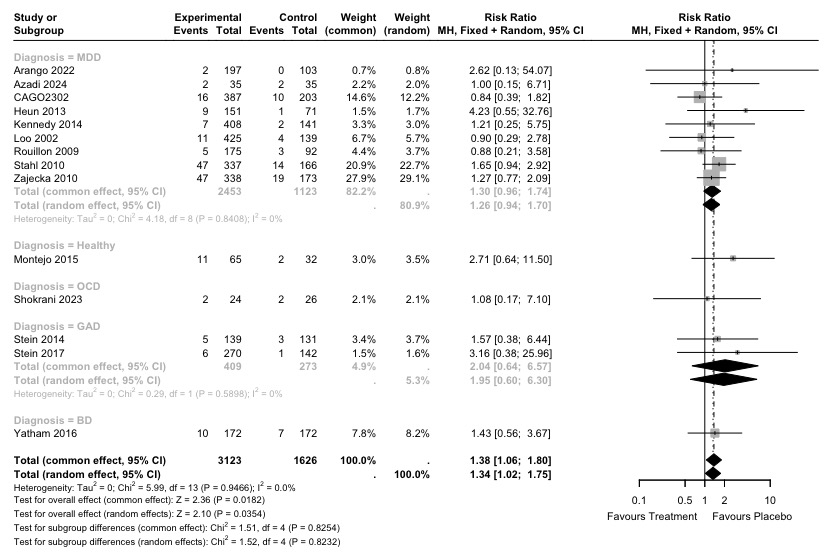
**

*Note.* RR = Risk Ratio for Somnolence as treatment emergent adverse effect with 95% CI (Confidence Intervals).

**Figure 7.3.** Forest plot – Somnolence as treatment emergent adverse effect, subgroup by use of add-on drug treatment.

**
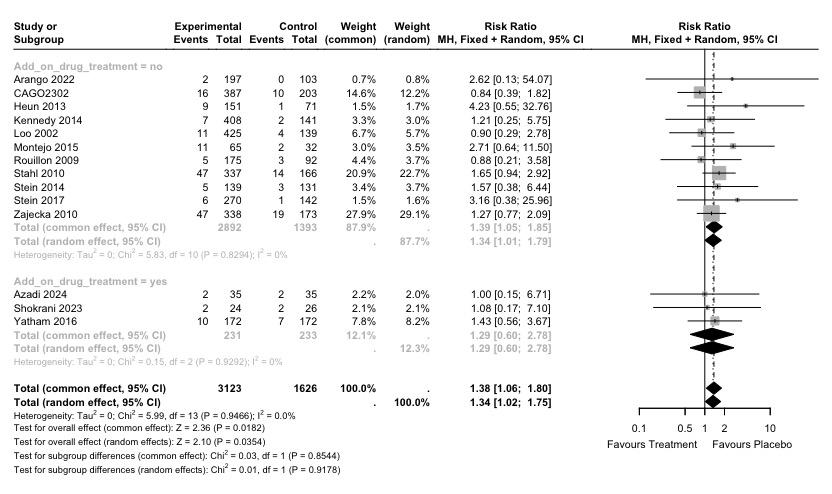
**

*Note.* RR = Risk Ratio for Somnolence as treatment emergent adverse effect with 95% CI (Confidence Intervals).

**Figure 7.4.** Forest plot - Somnolence as treatment emergent adverse effect, subgroup by age.

**
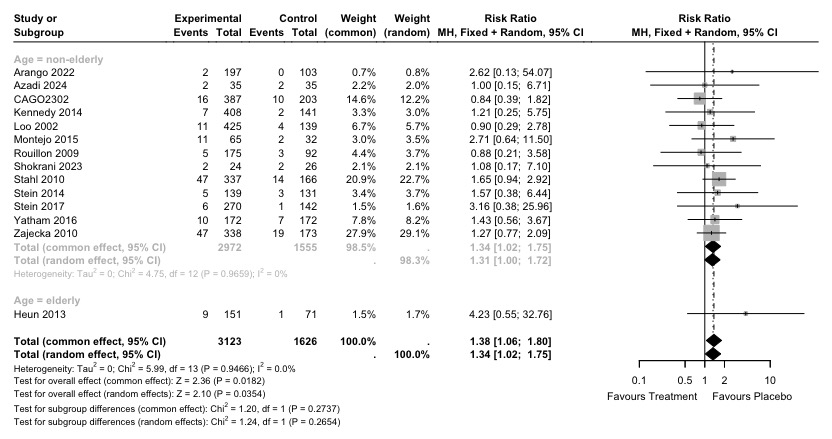
**

*Note.* RR = Risk Ratio for Somnolence as treatment emergent adverse effect with 95% CI (Confidence Intervals).

**Figure S7.5.** Forest plot - Somnolence as treatment emergent adverse effect, Sensitivity Analysis – excluding sponsored studies.

**
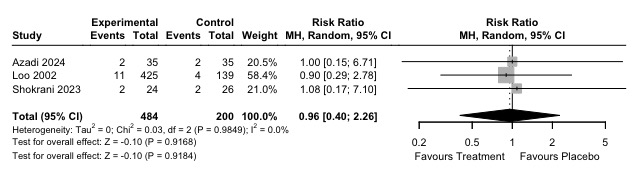
**

*Note.* RR = Risk Ratio for Somnolence as treatment emergent adverse effect with 95% CI (Confidence Intervals).

**Figure S7.6.** Forest plot - Somnolence as treatment emergent adverse effect, Sensitivity Analysis - excluding studies using hypnotics.

**
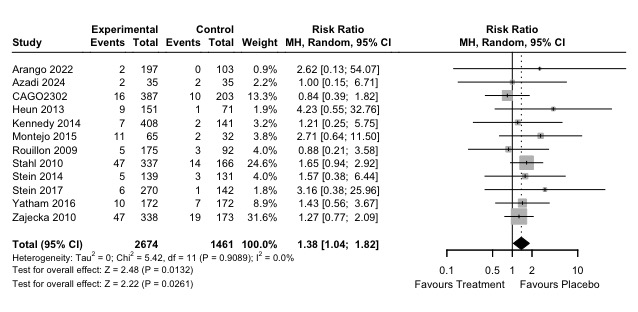
**

*Note.* RR = Risk Ratio for Somnolence as treatment emergent adverse effect with 95% CI (Confidence Intervals).

## **Secondary Outcomes**

### **Sleep onset latency (Getting to sleep score measured in LSEQ)**

**Figure S8.1.** Forest plot - Sleep onset latency (Getting off to sleep score measured in LSEQ), pooled result.


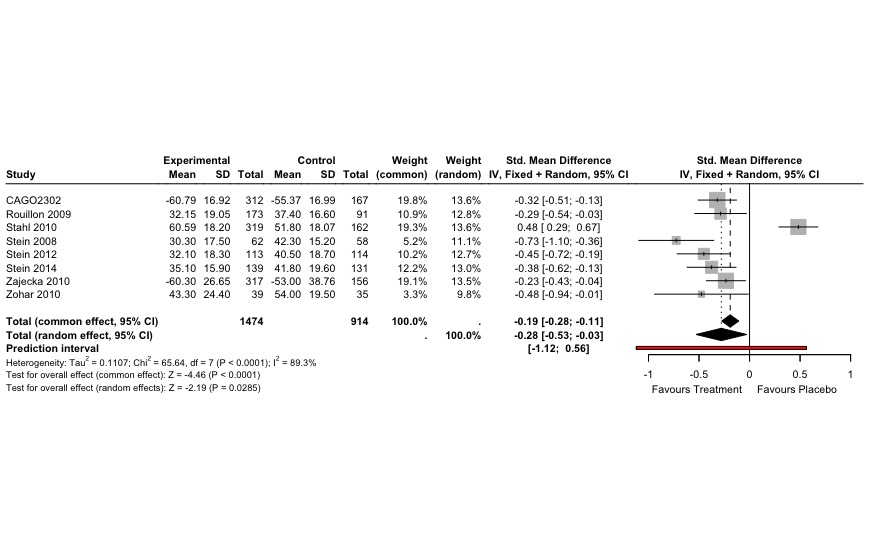


*Note.* SMD = standardized mean difference for Sleep onset latency (Getting off to sleep score measured in LSEQ) with 95% CI (Confidence Intervals) and PI (Prediction Intervals).

### **Sleep onset latency (minutes)**

**Figure S8.2.** Forest plot - Sleep onset latency (measured in minutes), pooled result.


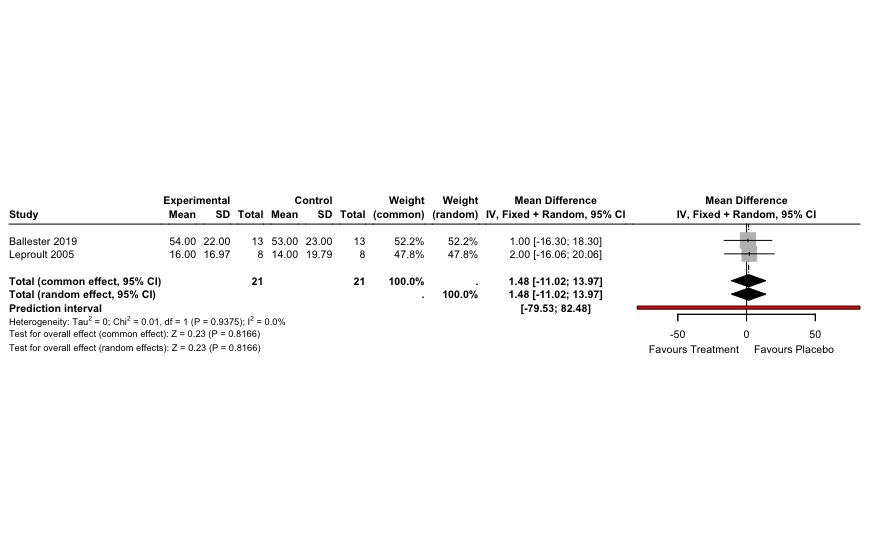


*Note.* MD = weighted mean difference for Sleep onset latency (measured in minutes) with 95% CI (Confidence Intervals) and PI (Prediction Intervals).

### **Number of nocturnal awakenings**

**Figure S9.** Forest plot - Number of nocturnal awakenings, pooled result.


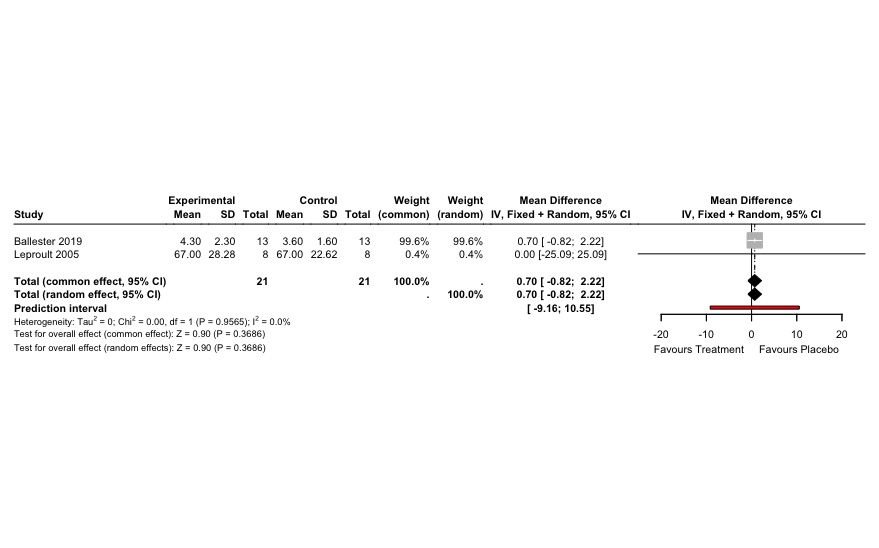


*Note.* MD = weighted mean difference for Number of nocturnal awakenings with 95% CI (Confidence Intervals) and PI (Prediction Intervals).

### **Nocturnal time spent awake after sleep onset**

**Figure S10.** Forest plot - Nocturnal time spent awake after sleep onset measured in minutes, pooled result.


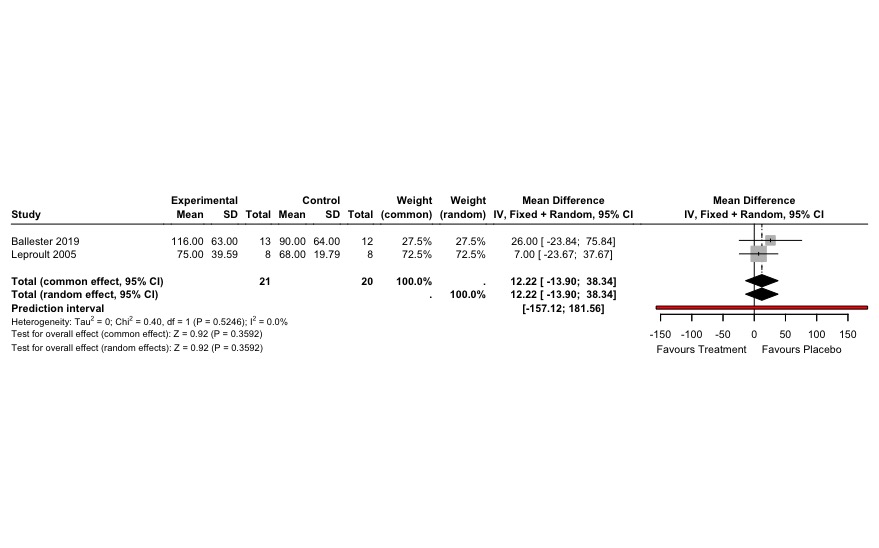


*Note.* MD = weighted mean difference for Nocturnal time spent awake after sleep onset with 95% CI (Confidence Intervals) and PI (Prediction Intervals).

### **Daytime impairment (Sleep awakening score)**

**Figure S11.** Forest plot - Sleep awakening score measured in LSEQ, pooled result.


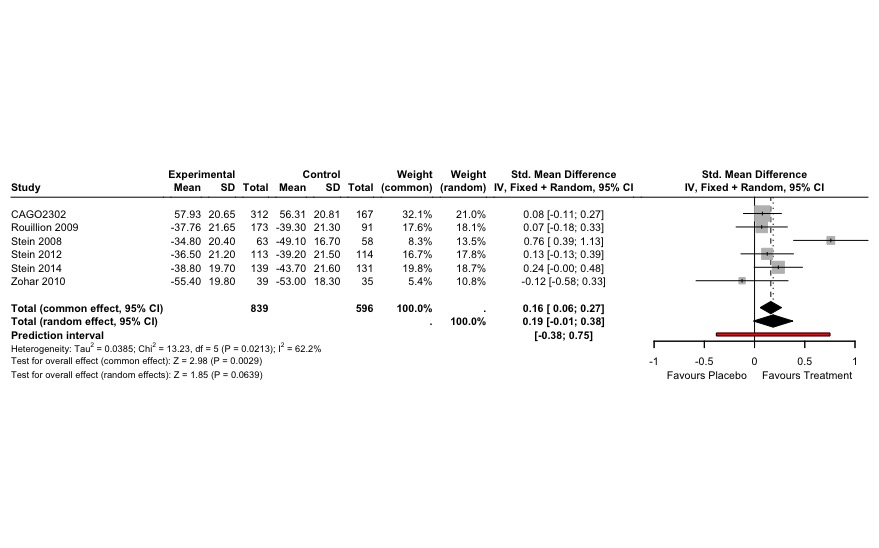


*Note.* SMD = standardized mean difference for Sleep awakening score measured in LSEQ with 95% CI (Confidence Intervals) and PI (Prediction Intervals).

### **Number of dropouts due to adverse effects**

**Figure S12.** Forest plot - Number of Dropouts due to Adverse Effects, pooled result.


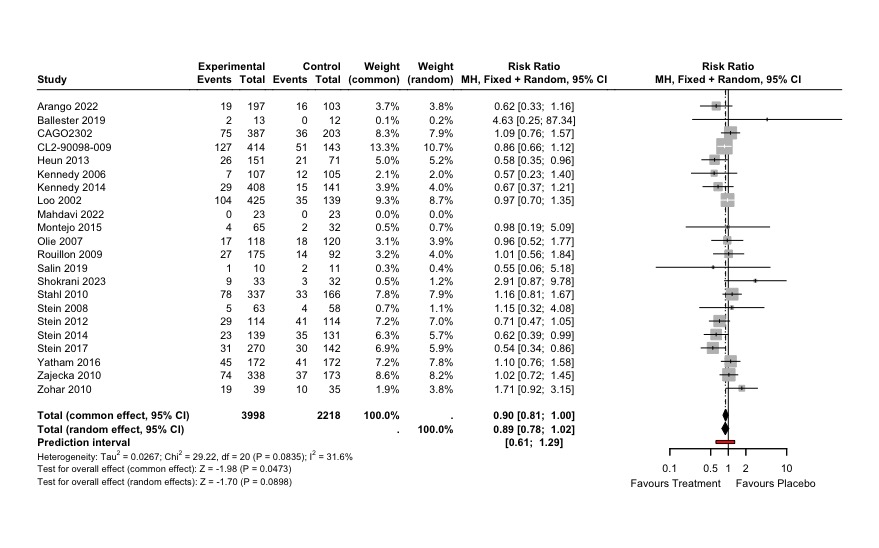


*Note.* RR = Risk Ratio for Number of Dropouts due to Adverse Effects with 95% CI (Confidence Intervals) and PI (Prediction Intervals).

### **Number of dropouts due to sleep-related adverse effects**

**Figure S13.** Forest plot - Number of Dropouts due to sleep-related adverse effects, pooled result.


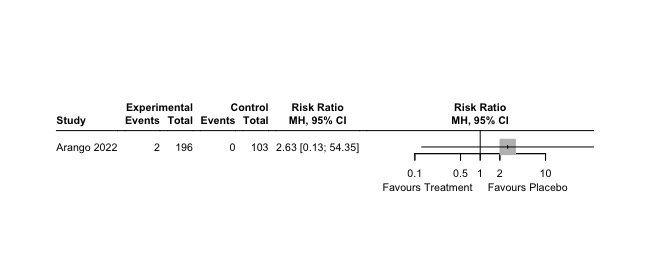


*Note.* RR = Risk Ratio for Number of Dropouts due to sleep-related adverse effects with 95% CI (Confidence Intervals).

### **Number of participants with adverse effects**

**Figure S14.** Forest plot – Number of participants with Adverse effects, pooled result.


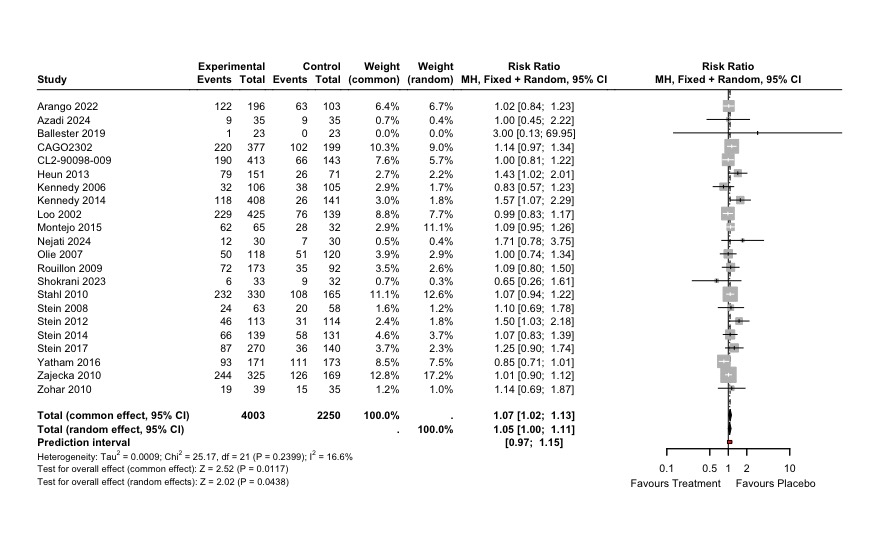


*Note.* RR = Risk Ratio for Number of participants with Adverse Effects with 95% CI (Confidence Intervals) and PI (Prediction Intervals).

### **Number of participants with sleep related adverse effects**

**Figure S15.** Forest plot – Number of participants with Sleep related Adverse Effects, pooled result.

**
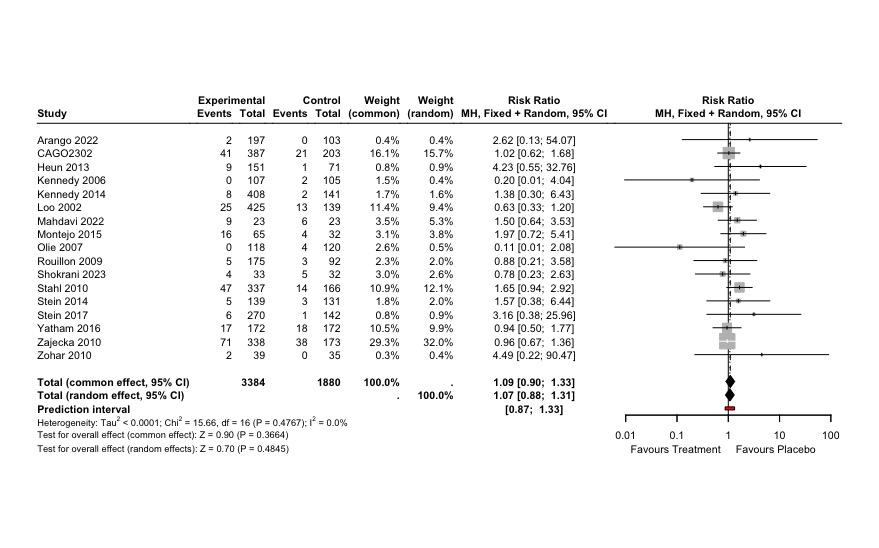
**

*Note.* RR = Risk Ratio for Number of participants with Sleep related Adverse Effects with 95% CI (Confidence Intervals) and PI (Prediction Intervals).

### **Other (Behaviour Integrity)**

**Figure S16.** Forest plot – Behaviour Integrity (LSEQ subscale), pooled result.

**
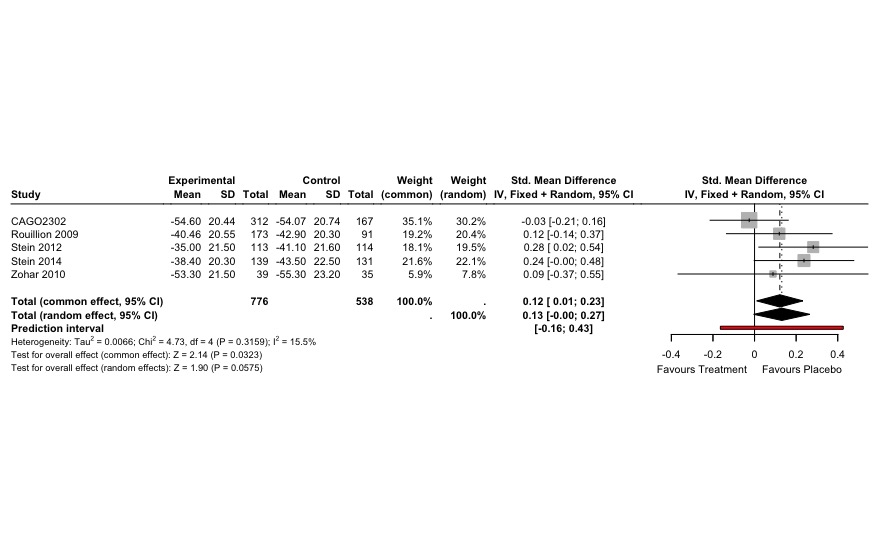
**

*Note.* SMD = Standardized Mean Difference for Behaviour Integrity (LSEQ subscale) with 95% CI (Confidence Intervals) and PI (Prediction Intervals).

# **Assessment of Publication bias**

## **Funnel plots: Primary outcomes with at least 10 studies**

### **Funnel plot: Somnolence as treatment emergent adverse effect**

**Figure S17.** Funnel plot of studies reporting Somnolence.

**
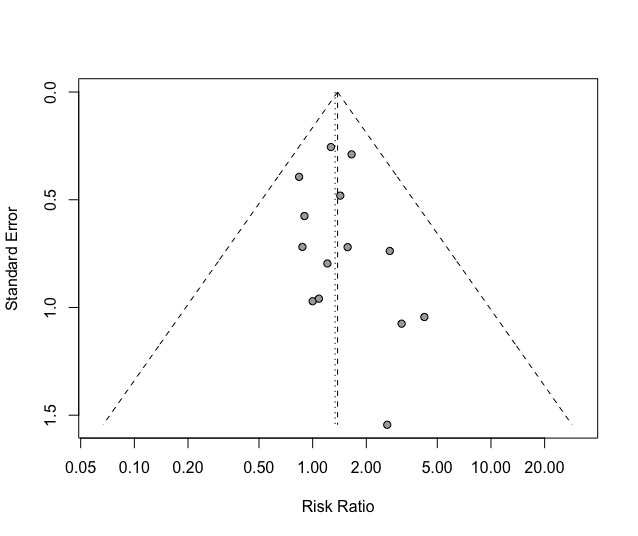
**

## **Trim-and- fill funnel plots: Primary outcomes with at least 10 studies**

### **Trim-and-fill plot: Somnolence as treatment emergent adverse effect**

**Figure S18.** Funnel plot of studies reporting Somnolence, after trim and fill method.

**
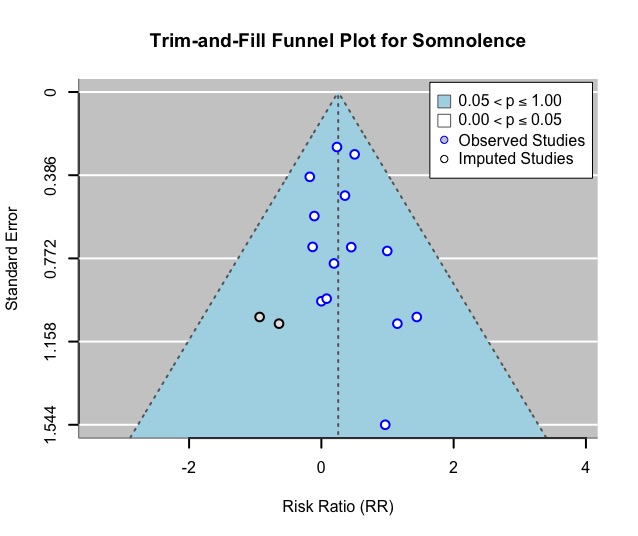
**

## **Egger’s regression test for funnel plot asymmetry: Primary outcomes with at least 10 studies**

### **Egger’s regression test for funnel plot asymmetry: Somnolence as treatment emergent adverse effect**

**Egger’s test**

Regression Test for Funnel Plot Asymmetry

Model: weighted regression with multiplicative dispersion

Predictor: standard error

Test for Funnel Plot Asymmetry: t = 0.8738, df = 12, p = 0.3994

Limit Estimate (as sei -> 0): b = 0.1419 (CI: -0.2804, 0.5641)

# **Head-to-head comparisons of agomelatine with other antidepressants**

**Table S6.** Head-to-head efficacy comparisons of agomelatine with other antidepressants

| **Antidepressant Compared** | **Author** | **Year** | **Diagnosis** | **Outcomes** | **Results** |
| --- | --- | --- | --- | --- | --- |
| Escitalopram | Quera-Salva et al. | 2013 | MDD | Polysomnographic and subjective | No difference |
|  | Corruble et al. | 2013 | MDD | Polysomnographic and subjective | No difference |
|  | Stein et al. | 2018 | GAD | Polysomnographic and subjective | No difference |
| Sertraline | Kasper et al. | 2010 | MDD | Actigraphic | Favors Agomelatine |
|  |  |  |  | Subjective | No difference |
| Fluoxetine | Shu et al. | 2014 | MDD | Subjective | No difference |
| Mirtazapine | Mi et al. | 2020 | MDD | Polysomnographic and subjective | No difference |
| Venlafaxine | Lemoine et al. | 2007 | MDD | Subjective | Favors Agomelatine |
| Duloxetine | Marey & Servier Laboratories | 2020 | MDD | Subjective | No difference |
| **Abbreviations:**  **GAD:** Generalized Anxiety Disorder; **MDD:** Major Depressive Disorder | | | | | |

# **References**

Arango, C., Buitelaar, J. K., Fegert, J. M., Olivier, V., Pénélaud, P.-F., Marx, U., Chimits, D., Falissard, B., Barylnik, J., Birdeanu, L., Bosch, G. P., Boychevskaya, J., Boyev, I., Bugán, E., Bukhanovskaya, O., Chaban, O., Dobrescu, I., Feller, G., Flisiak-Antonijczuk, H., … Wolañczyk, T. (2022). Safety and efficacy of agomelatine in children and adolescents with major depressive disorder receiving psychosocial counselling: A double-blind, randomised, controlled, phase 3 trial in nine countries. *The Lancet Psychiatry*, *9*(2), 113–124. <https://doi.org/10.1016/S2215-0366(21)00390-4>

Azadi, H., Rashidpour, P., Yassini Ardekani, S. M., Nadi Sakhvidi, M., Afshang, H., & Bidaki, R. (2024). The Effect of Adding Agomelatine  to Escitalopram in the Treatment of Major Depressive Disorder. *Nevrologiya, Neiropsikhiatriya, Psikhosomatika = Neurology, Neuropsychiatry,  Psychosomatics*, *16*(5), 24–29. <https://doi.org/10.14412/2074-2711-2024-5-24-29>

Ballester, P., Martínez, M. J., Inda, M.-M., Javaloyes, A., Richdale, A. L., Muriel, J., Belda, C., Toral, N., Morales, D., Fernández, E., & Peiró, A. M. (2019). Evaluation of agomelatine for the treatment of sleep problems in adults with autism spectrum disorder and co-morbid intellectual disability. *Journal of Psychopharmacology*, *33*(11), 1395–1406. <https://doi.org/10.1177/0269881119864968>

ChiCTR1800018822. (2018). *A randomized, double-blind, controlled trial for investigation of the improvement of agomelatine combined cognitive behavioral therapy in patients with chronic insomnia with mild depression* [Trial Registry]. Chinese Clinical Trial Registry. <https://www.chictr.org.cn/showproj.aspx?proj=30321>

ChiCTR2000032518. (2020). *Efficacy and safety of agomelatine combined with SSRIs in depressive disorder: A randomized controlled trial* [Trial Registry]. Chinese Clinical Trial Registry. <https://www.chictr.org.cn/showprojEN.html?proj=50671>

ChiCTR2100046289. (2021). *Efficacy and safety of agomelatine in patients epilepsy coexisting with depression on mood and cognitive function* [Trial Registry]. Chinese Clinical Trial Registry. <https://www.chictr.org.cn/showprojEN.html?proj=126182>

ChiCTR2100048875. (2022). *A multicenter, randomized, double-blind, placebo-controlled trial of the efficacy and safety of agomelatine in patients with rapid eye movement (REM) sleep behavior disorder (RBD)* [Trial Registry]. Chinese Clinical Trial Registry. <https://www.chictr.org.cn/showprojEN.html?proj=130377>

CL2-90098-009. (n.d.). *Efficacy and safety of 2 doses of S 90098 (1 and 2mg/day), sublingual formulation for 8 weeks in out-patients with Major Depressive Disorder. An 8-week randomised, double-blind, fixed dose, international, multicentre, placebo-controlled study with parallel groups, followed by an extension double-blind treatment period for 16 weeks.* Servier Clinical Trials. Retrieved December 27, 2024, from <https://clinicaltrials.servier.com/trial/efficacy-and-safety-of-2-doses-of-s-90098-1-and-2mgday-sublingual-formulation-for-8-weeks-in-out-patients-with-major-depressive-disorder-an-8-week-randomised-double-blind-fixed-dose-internatio/>

Corruble, E., de Bodinat, C., Belaïdi, C., Goodwin, G. M., & agomelatine study group. (2013). Efficacy of agomelatine and escitalopram on depression, subjective sleep and emotional experiences in patients with major depressive disorder: A 24-wk randomized, controlled, double-blind trial. *The International Journal of Neuropsychopharmacology*, *16*(10), 2219–2234. <https://doi.org/10.1017/S1461145713000679>

CTRI/2011/08/001946. (2011). *A Multicentric, Open-label, Randomized, Comparative, Parallel-group, Active-Controlled Phase III Clinical Trial of the Efficacy and Safety of Agomelatine oral tablets in patients with Major Depressive Disorder* [Trial Registry]. Clinical Trials Registry - India (CTRI). <https://www.ctri.nic.in/Clinicaltrials/pmaindet2.php?EncHid=MjU2OQ==&Enc=&userName=agomelatine>

Dehghan Nayeri, N., Farzin, K., Shafiee Sabet, M., & Tafakhori, A. (2024). Study Protocol for Assessing the Effectiveness of Agomelatine on the Severity and Frequency of Episodic Migraine Attacks Without Aura: A Randomized Triple-Blind, Placebo-Controlled Trial. *Shiraz E-Medical Journal*, *25*(6). <https://doi.org/10.5812/semj-142956>

EUCTR2013-003370-27-DE. (2014). *Functional MRI (fMRI) after challenge and treatment with antidepressants and their relation to the clinical course, to the Hypothalamus-Hypophysis-Adrenocortical Axis and the colon microbiome* [Trial Registry]. EU Clinical Trials Register. <https://www.clinicaltrialsregister.eu/ctr-search/search?query=EUCTR2013-003370-27-DE>

Furukawa, T. A., Salanti, G., Atkinson, L. Z., Leucht, S., Ruhe, H. G., Turner, E. H., Chaimani, A., Ogawa, Y., Takeshima, N., Hayasaka, Y., Imai, H., Shinohara, K., Suganuma, A., Watanabe, N., Stockton, S., Geddes, J. R., & Cipriani, A. (2016). Comparative efficacy and acceptability of first-generation and second-generation antidepressants in the acute treatment of major depression: Protocol for a network meta-analysis. *BMJ Open*, *6*(7), e010919. <https://doi.org/10.1136/bmjopen-2015-010919>

Heun, R., Ahokas, A., Boyer, P., Giménez-Montesinos, N., Pontes-Soares, F., Olivier, V., & Group, on B. of the A. S. (2013). The Efficacy of Agomelatine in Elderly Patients With Recurrent Major Depressive Disorder: A Placebo-Controlled Study. *The Journal of Clinical Psychiatry*, *74*(6), 5943. <https://doi.org/10.4088/JCP.12m08250>

IRCT20170608034390N10. (2022). *Evaluation of the Efficacy of Agomelatine as Add-on Therapy on the Aggression, Agitation, and its Relationship with Serum Brain-Derived Neurotrophic Factor (BDNF) of Patients with Alzheimer’s disease Dementia; A Double-blind, Randomized, Placebo-controlled Clinical Trial* [Trial Registry]. International Clinical Trials Registry Platfom (ICTRP). <https://trialsearch.who.int/Trial2.aspx?TrialID=IRCT20170608034390N10>

IRCT20220508054780N4. (2024). *A clinical trial to compare the effectiveness of agomelatine with placebo on rate of delirium in patients admitted to the cardiac surgery intensive care unit* [Trial Registry]. International Clinical Trials Registry Platfom (ICTRP). <https://trialsearch.who.int/Trial2.aspx?TrialID=IRCT20220508054780N4>

IRCT20230303057599N1. (2023). *The effectiveness of agomelatine on the severity and numbers of migraine attacks in patients with migraine* [Trial Registry]. International Clinical Trials Registry Platfom (ICTRP). <https://trialsearch.who.int/Trial2.aspx?TrialID=IRCT20230303057599N1>

Kennedy, S. H., Avedisova, A., Giménez-Montesinos, N., Belaïdi, C., de Bodinat, C., & Agomelatine Study Group. (2014). A placebo-controlled study of three agomelatine dose regimens (10 mg, 25 mg, 25-50 mg) in patients with major depressive disorder. *European Neuropsychopharmacology: The Journal of the European College of Neuropsychopharmacology*, *24*(4), 553–563. <https://doi.org/10.1016/j.euroneuro.2014.01.006>

Kennedy, S. H., & Emsley, R. (2006). Placebo-controlled trial of agomelatine in the treatment of major depressive disorder. *European Neuropsychopharmacology: The Journal of the European College of Neuropsychopharmacology*, *16*(2), 93–100. <https://doi.org/10.1016/j.euroneuro.2005.09.002>

Lemoine, P., Guilleminault, C., & Alvarez, E. (2007). Improvement in subjective sleep in major depressive disorder with a novel antidepressant, agomelatine: Randomized, double-blind comparison with venlafaxine. *The Journal of Clinical Psychiatry*, *68*(11), 1723–1732. <https://doi.org/10.4088/jcp.v68n1112>

Leproult, R., Van Onderbergen, A., L’Hermite-Balériaux, M., Van Cauter, E., & Copinschi, G. (2005). Phase-shifts of 24-h rhythms of hormonal release and body temperature following early evening administration of the melatonin agonist agomelatine in healthy older men. *Clinical Endocrinology*, *63*(3), 298–304. <https://doi.org/10.1111/j.1365-2265.2005.02341.x>

Lôo, H., Hale, A., & D’haenen, H. (2002). Determination of the dose of agomelatine, a melatoninergic agonist and selective 5-HT2C antagonist, in the treatment of major depressive disorder: A placebo-controlled dose range study. *International Clinical Psychopharmacology*, *17*(5), 239.

Mahdavi, S. M., Shariati, B., Shalbafan, M., Rashedi, V., Yarahmadi, M., Ghaznavi, A., & Amiri, S. (2022). The effectiveness of pregabalin with or without agomelatine in the treatment of chronic low back pain: A double-blind, placebo-controlled, randomized clinical trial. *BMC Pharmacology & Toxicology*, *23*(1), 70. <https://doi.org/10.1186/s40360-022-00612-3>

Marey, C., & Servier Laboratories. (2020). *Evaluation of efficacy and clinical benefit of agomelatine in patients with major depressive disorder compared to serotonin-norepinephrine reuptake inhibitor (SNRI)*. ISRCTN Registry. <https://doi.org/10.1186/ISRCTN96725312>

Mi, W.-F., Tabarak, S., Wang, L., Zhang, S.-Z., Lin, X., Du, L.-T., Liu, Z., Bao, Y.-P., Gao, X.-J., Zhang, W.-H., Wang, X.-Q., Fan, T.-T., Li, L.-Z., Hao, X.-N., Fu, Y., Shi, Y., Guo, L.-H., Sun, H.-Q., Liu, L., … Li, S.-X. (2020). Effects of agomelatine and mirtazapine on sleep disturbances in major depressive disorder: Evidence from polysomnographic and resting-state functional connectivity analyses. *Sleep*, *43*(11), zsaa092. <https://doi.org/10.1093/sleep/zsaa092>

Montejo, A. L., Deakin, J., Gaillard, R., Harmer, C., Meyniel, F., Jabourian, A., Gabriel, C., Gruget, C., Klinge, C., MacFayden, C., Milligan, H., Mullings, E., & Goodwin, G. (2015). Better sexual acceptability of agomelatine (25 and 50 mg) compared to escitalopram (20 mg) in healthy volunteers. A 9-week, placebo-controlled study using the PRSexDQ scale. *Journal of Psychopharmacology*, *29*(10), 1119–1128. <https://doi.org/10.1177/0269881115599385>

NCT01110902. (2020). *A 8-week, Randomized, Double-blind, Placebo-controlled, Parallel-group, Multi-center Study of the Efficacy and Safety of Agomelatine 0.5 mg and 1 mg Sublingual Tablets Administered Once Daily in Patients With Major Depressive Disorder (MDD)* (Clinical Trial Registration CAGO178C2302). Novartis Pharmaceuticals. <https://clinicaltrials.gov/study/NCT01110902>

NCT04589143. (2024). *A Double-blind, Placebo-controlled Study of Antidepressant Augmentation With Agomelatine* [Trial Registry]. Clinical Trials. <https://clinicaltrials.gov/study/NCT04589143>

NCT05426304. (2022). *Prophylactic Effects of Agomelatine for Poststroke Depression (PRAISED)* [Trial Registry]. Clinical Trials. <https://clinicaltrials.gov/study/NCT05426304>

Nejati, A., Bazrafshan, A., & Mosavat, S. H. (2024). Agomelatine efficacy in treatment resistant obsessive-compulsive disorder: A randomized controlled trial. *International Journal of Psychiatry in Medicine*, *59*(5), 545–555. <https://doi.org/10.1177/00912174231225763>

Olié, J. P., & Kasper, S. (2007). Efficacy of agomelatine, a MT1/MT2 receptor agonist with 5-HT2C antagonistic properties, in major depressive disorder. *International Journal of Neuropsychopharmacology*, *10*(5), 661–673. <https://doi.org/10.1017/S1461145707007766>

Quera-Salva, M.-A., Hajak, G., Philip, P., Montplaisir, J., Keufer-Le Gall, S., Laredo, J., & Guilleminault, C. (2011). Comparison of agomelatine and escitalopram on nighttime sleep and daytime condition and efficacy in major depressive disorder patients. *International Clinical Psychopharmacology*, *26*(5), 252–262. <https://doi.org/10.1097/YIC.0b013e328349b117>

Rouillon, F., & Servier Laboratories. (2008). *Efficacy and safety of two doses of S 90098 (1 and 2 mg/day), sublingual formulation for 8 weeks in out‐patients with major depressive disorder: An 8‐week randomised, double‐blind, fixed dose, international, multicentre, placebo‐controlled study with parallel groups, followed by an extension double‐blind treatment period for 16 weeks* (Controlled‐Trials.Com [ISRCTN38378163; CL2‐90098‐005]). [[www.controlled‐trials.com]](https://doi.org/%5bwww.controlled‐trials.com%5d)

Salin, K., Kasitanon, N., Maneeton, B., & Louthrenoo, W. (2019, April 25). *The Effect of Agomelatine on Sleep Disturbance, Depression, and Anxiety in Patients with Systemic Lupus Erythematosus: A Randomized, DoubleBlinded Placebo-Controlled Trial*. The 35th Annual Meeting the Royal College of Physicians of Thailand “Towards Better and Safer Patient Care,” Chonburi, PEACH Royal Cliff Beach Resort, Pattaya, Thailand.

Shokrani, M., Askari, S., Eissazade, N., Shariat, S. V., Shariati, B., Yarahmadi, M., & Shalbafan, M. (2023). Agomelatine augmentation of sertraline in the treatment of moderate to severe obsessive-compulsive disorder: A randomized double-blinded placebo-controlled clinical trial. *BMC Psychiatry*, *23*(1), 686. <https://doi.org/10.1186/s12888-023-05189-7>

Shu, L., Sulaiman, A. H., Huang, Y. S., Fones Soon Leng, C., Crutel, V. S., & Kim, Y. S. (2014). Comparable efficacy and safety of 8 weeks treatment with agomelatine 25-50mg or fluoxetine 20-40mg in Asian out-patients with major depressive disorder. *Asian Journal of Psychiatry*, *8*, 26–32. <https://doi.org/10.1016/j.ajp.2013.09.009>

Stahl, S., Fava, M., Trivedi, M. H., Caputo, A., Shah, A., & Post, A. (2010). Agomelatine in the Treatment of Major Depressive Disorder: An 8-Week, Multicenter, Randomized, Placebo-Controlled Trial. *The Journal of Clinical Psychiatry*, *71*(5), 669. <https://doi.org/10.4088/JCP.09m05471blu>

Stein, D. J., Ahokas, A. A., & de Bodinat, C. (2008). Efficacy of Agomelatine in Generalized Anxiety Disorder: A Randomized, Double-Blind, Placebo-Controlled Study. *Journal of Clinical Psychopharmacology*, *28*(5), 561. <https://doi.org/10.1097/JCP.0b013e318184ff5b>

Stein, D. J., Ahokas, A., Albarran, C., Olivier, V., & Allgulander, C. (2012). Agomelatine Prevents Relapse in Generalized Anxiety Disorder: A 6-Month Randomized, Double-Blind, Placebo-Controlled Discontinuation Study. *The Journal of Clinical Psychiatry*, *73*(7), 665. <https://doi.org/10.4088/JCP.11m07493>

Stein, D. J., Ahokas, A., Jarema, M., Avedisova, A. S., Vavrusova, L., Chaban, O., Gruget, C., Olivier, V., Picarel-Blanchot, F., & de Bodinat, C. (2017). Efficacy and safety of agomelatine (10 or 25 mg/day) in non-depressed out-patients with generalized anxiety disorder: A 12-week, double-blind, placebo-controlled study. *European Neuropsychopharmacology*, *27*(5), 526–537. <https://doi.org/10.1016/j.euroneuro.2017.02.007>

Stein, D. J., Ahokas, A., Márquez, M. S., Höschl, C., Oh, K. S., Jarema, M., Avedisova, A. S., Albarran, C., & Olivier, V. (2014). Agomelatine in Generalized Anxiety Disorder: An Active Comparator and Placebo-Controlled Study. *The Journal of Clinical Psychiatry*, *75*(4), 663. <https://doi.org/10.4088/JCP.13m08433>

Stein, D. J., Khoo, J.-P., Ahokas, A., Jarema, M., Van Ameringen, Michael., Vavrusova, L., Hӧschl, C., Bauer, M., Bitter, I., Mosolov, S. N., Olivier, V., Matharan, S., Picarel-Blanchot, F., & de Bodinat, C. (2018). 12-week double-blind randomized multicenter study of efficacy and safety of agomelatine (25–50 mg/day) *versus* escitalopram (10–20 mg/day) in out-patients with severe generalized anxiety disorder. *European Neuropsychopharmacology*, *28*(8), 970–979. <https://doi.org/10.1016/j.euroneuro.2018.05.006>

Yatham, L. N., Vieta, E., Goodwin, G. M., Bourin, M., Bodinat, C. de, Laredo, J., & Calabrese, J. (2016). Agomelatine or placebo as adjunctive therapy to a mood stabiliser in bipolar I depression: Randomised double-blind placebo-controlled trial. *The British Journal of Psychiatry*, *208*(1), 78–86. <https://doi.org/10.1192/bjp.bp.114.147587>

Zajecka, J., Schatzberg, A., Stahl, S., Shah, A., Caputo, A., & Post, A. (2010). Efficacy and Safety of Agomelatine in the Treatment of Major Depressive Disorder: A Multicenter, Randomized, Double-Blind, Placebo-Controlled Trial. *Journal of Clinical Psychopharmacology*, *30*(2), 135. <https://doi.org/10.1097/JCP.0b013e3181d420a7>

Zohar, & Servier Laboratories. (2009). *Efficacy of agomelatine 25 mg/day (with possible increase to 50 mg/day after 8 weeks of treatment) given orally during 16 weeks in patients with Obsessive-Compulsive Disorder.A randomised, double-blind, placebo-controlled, parallel groups, international study.* ([CL2-20098-072;EUCTR2009-016713-20]. EU Clinical Trials Register). [www.clinicaltrialsregister.eu](https://doi.org/www.clinicaltrialsregister.eu)
